# Automated detection of cerebral microbleeds on MR images using knowledge distillation framework

**DOI:** 10.3389/fninf.2023.1204186

**Published:** 2023-07-10

**Authors:** Vaanathi Sundaresan, Christoph Arthofer, Giovanna Zamboni, Andrew G. Murchison, Robert A. Dineen, Peter M. Rothwell, Dorothee P. Auer, Chaoyue Wang, Karla L. Miller, Benjamin C. Tendler, Fidel Alfaro-Almagro, Stamatios N. Sotiropoulos, Nikola Sprigg, Ludovica Griffanti, Mark Jenkinson

**Affiliations:** ^1^Department of Computational and Data Sciences, Indian Institute of Science, Bengaluru, Karnataka, India; ^2^Wellcome Centre for Integrative Neuroimaging, Oxford Centre for Functional MRI of the Brain, Nuffield Department of Clinical Neurosciences, University of Oxford, Oxford, United Kingdom; ^3^National Institute for Health and Care Research (NIHR) Nottingham Biomedical Research Centre, Queen's Medical Centre, University of Nottingham, Nottingham, United Kingdom; ^4^Sir Peter Mansfield Imaging Centre, University of Nottingham, Nottingham, United Kingdom; ^5^Centre for Prevention of Stroke and Dementia, Nuffield Department of Clinical Neurosciences, University of Oxford, Oxford, United Kingdom; ^6^Dipartimento di Scienze Biomediche, Metaboliche e Neuroscienze, Universitá di Modena e Reggio Emilia, Modena, Italy; ^7^Department of Neuroradiology, Oxford University Hospitals National Health Service (NHS) Foundation Trust, Oxford, United Kingdom; ^8^Radiological Sciences, Mental Health and Clinical Neurosciences, School of Medicine, University of Nottingham, Nottingham, United Kingdom; ^9^Stroke Trials Unit, Mental Health and Clinical Neuroscience, University of Nottingham, Nottingham, United Kingdom; ^10^Wellcome Centre for Integrative Neuroimaging, Oxford Centre for Human Brain Activity, Department of Psychiatry, University of Oxford, Oxford, United Kingdom; ^11^South Australian Health and Medical Research Institute, Adelaide, SA, Australia; ^12^Australian Institute for Machine Learning, School of Computer Science, The University of Adelaide, Adelaide, SA, Australia

**Keywords:** deep learning, knowledge distillation, detection, susceptibility-weighted image (SWI), quantitative susceptibility mapping (QSM), magnetic resonance imaging, cerebral microbleed (CMB)

## Abstract

**Introduction:**

Cerebral microbleeds (CMBs) are associated with white matter damage, and various neurodegenerative and cerebrovascular diseases. CMBs occur as small, circular hypointense lesions on T2*-weighted gradient recalled echo (GRE) and susceptibility-weighted imaging (SWI) images, and hyperintense on quantitative susceptibility mapping (QSM) images due to their paramagnetic nature. Accurate automated detection of CMBs would help to determine quantitative imaging biomarkers (e.g., CMB count) on large datasets. In this work, we propose a fully automated, deep learning-based, 3-step algorithm, using structural and anatomical properties of CMBs from any single input image modality (e.g., GRE/SWI/QSM) for their accurate detections.

**Methods:**

In our method, the first step consists of an initial candidate detection step that detects CMBs with high sensitivity. In the second step, candidate discrimination step is performed using a knowledge distillation framework, with a multi-tasking teacher network that guides the student network to classify CMB and non-CMB instances in an offline manner. Finally, a morphological clean-up step further reduces false positives using anatomical constraints. We used four datasets consisting of different modalities specified above, acquired using various protocols and with a variety of pathological and demographic characteristics.

**Results:**

On cross-validation within datasets, our method achieved a cluster-wise true positive rate (TPR) of over 90% with an average of <2 false positives per subject. The knowledge distillation framework improves the cluster-wise TPR of the student model by 15%. Our method is flexible in terms of the input modality and provides comparable cluster-wise TPR and better cluster-wise precision compared to existing state-of-the-art methods. When evaluating across different datasets, our method showed good generalizability with a cluster-wise TPR >80 % with different modalities. The python implementation of the proposed method is openly available.

## 1. Introduction

Cerebral microbleeds (CMBs) are haemosiderin deposits due to micro-hemorrhages in the brain. CMBs are found in subjects with cerebrovascular diseases, cognitive impairment and dementia, and also found in healthy elderly subjects. CMBs have been associated with white matter damage, and various neurogenerative diseases including Alzheimer's disease and cerebral amyloid angiopathy (CAA). The presence of CMBs has also been shown to increase the risk of symptomatic intracerebral hemorrhage (ICH) and stroke (Cordonnier et al., 2007). Identification of CMBs and determining their distribution could help in obtaining important biomarkers for various diseases [e.g., lobar CMBs and deep/infratentorial CMBs might indicate CAA and hypertensive vasculopathy, respectively (Greenberg et al., 2009)].

CMBs appear as small, circular, well-defined hypointense lesions ranging from 2 to 10 mm in size on T2*-weighted gradient recalled echo (GRE) images. Due to the paramagnetic susceptibility of the iron content in the CMBs, modalities such as susceptibility-weighted imaging (SWI) (Haacke et al., 2004) and quantitative susceptibility mapping (QSM) images (Liu et al., 2015) are useful in the identification of CMBs. While all the above modalities are derived from the same scan, they use different aspects of data—T2*-weighted GRE are derived from magnitude only, QSM and SWI are derived from a combination of phase and magnitude. When compared to T2*-weighted GRE (T2*-GRE) images, CMBs appear more prominently on SWI images due to the blooming effect (Greenberg et al., 2009; Charidimou and Werring, 2011). Unlike T2*-GRE and SWI modalities, CMBs appear hyperintense on QSM images.

Automated detection of CMBs is highly challenging due to their small size, contrast variations, sparse distribution and the presence of imaging artefacts (e.g., ringing effect, susceptibility artefacts at tissue interfaces). Additionally, the presence of various “CMB-like” structures (or *mimics*) with diamagnetic (e.g., calcifications) and paramagnetic (e.g., micrometastases and hemorrhages) properties make the accurate detections of CMBs very difficult (for the list of mimics and their description, refer to Greenberg et al. (2009)). While the use of SWI images generally improves the CMB contrast when compared to GRE magnitude images (Nandigam et al., 2009; Shams et al., 2015), SWI also enhances mimics with magnetic susceptibility differences (both diamagnetic and paramagnetic), making it difficult to identify true CMBs (Greenberg et al., 2009). QSM could be useful to accurately identify true CMBs since it allows to separate diamagnetic tissues (with negative susceptibility, appearing hypointense) from paramagnetic tissues (with positive susceptibility, appearing hyperintense). On QSM images, CMBs appear hyperintense while diamagnetic mimics (e.g., calcifications) will appear hypointense (Rashid et al., 2021).

### 1.1. Existing literature on CMB detection

Various semi-automated and automated methods have been proposed for CMB detection. Most of the methods follow a common pattern with two steps: CMB candidate detection and post-processing to remove false positives (FPs). The first step generally achieves high sensitivity, while the second step is more challenging and leads to improvement in the precision. In the semi-automated methods, manual intervention has often been used in the cleaning-up step to remove FPs (Barnes et al., 2011; Seghier et al., 2011; van den Heuvel et al., 2016; Morrison et al., 2018). Occasionally, candidate detection (De Bresser et al., 2013; Lu et al., 2021a) and ground truth verification (Kuijf et al., 2012, 2013) also involve manual intervention. Manual detection of CMB candidates is extremely labor-intensive, especially when done on a large number of subjects (e.g., around 8,000 subjects from the UK Biobank Lu et al., 2021a), and might increase the risk of observer error, given the large number of scans and low prevalence rate. Fully automated methods, with high accuracy, could therefore be useful. Various fully automated methods have been proposed, with the candidate detection step often using hand-crafted shapes (Bian et al., 2013; Fazlollahi et al., 2014), intensity (Fazlollahi et al., 2015) and geometric features (Fazlollahi et al., 2014) within supervised classifier frameworks (Pan et al., 2008; Ghafaryasl et al., 2012; Fazlollahi et al., 2014, 2015; Dou et al., 2015). The FP reduction stage is typically based on supervised classifiers (Pan et al., 2008; Dou et al., 2015; Fazlollahi et al., 2015) using local intensity features and shape descriptors [e.g., Hessian-based shape descriptors (Fazlollahi et al., 2015)]. Among the shape descriptors, the radial symmetry transform has been most commonly used (Bian et al., 2013; Liu et al., 2019b), exploiting the circular shape of CMBs. Hence, using structural (e.g., intensity and shape) and anatomical information in combination with the local characteristics (e.g., local contrast) could aid in the reduction of FPs and more accurate detections of CMBs (Dou et al., 2015).

Conventional machine learning (ML) methods require the extraction of meaningful features capable of distinguishing CMBs from the background and mimics. However, due to the small size and variation in shape and intensities of CMBs, designing robust, descriptive and cost-effective features is highly challenging. The use of deep learning models, especially convolutional neural networks (CNNs) could overcome this challenge and provide more accurate CMB detection, since they efficiently extract both local and global contextual information. For instance, 3D CNN models have been used for feature extraction (Chen et al., 2015) and patch-level CMB detection (Dou et al., 2016). Dou et al. (2016) used a local region-based approach for the segmentation of CMB candidates and discrimination of CMB and non-CMB patches. They initially trained a 3D CNN with true CMB samples and randomly selected background samples. They then applied the initial model on the training set and used the false positive patches for enlarging the training dataset in the discrimination step. Another region-based CNN method using You Only Look Once (YOLO) (Redmon and Farhadi, 2017) was proposed by Al-Masni et al. (2020) (using a 3D CNN for FP reduction). In addition to the above methods, deep ResNets (He et al., 2016) were used for patch-level CMB classification (Chen et al., 2018; Liu et al., 2019b), along with a post-processing step using intensity morphological operations (Liu et al., 2019b). Given the size and sparsity of CMBs, class imbalance between CMBs and background is one of the major problems. Due to this, several methods used equal numbers of CMB patches and non-CMB patches, selected using manually annotated CMB voxels (and a comparable number of non-CMB voxels) for training and evaluation purposes (Zhang et al., 2016, 2018; Wang et al., 2019; Hong et al., 2020; Lu et al., 2021b). Note that patches selected in these methods may contain multiple CMBs.

### 1.2. Existing literature on knowledge distillation

Deep neural networks have been rapidly developing over recent years for accurate medical image segmentation tasks, including CMB segmentation, as mentioned above. However, the improved performance is achieved at the cost of long training times and using resource-intensive complex models (Lan et al., 2018). Hence, training small networks that are computationally efficient and generalizable across datasets is highly desirable. With this aim of model compression (Buciluçž et al., 2006), knowledge distillation (KD) (Ba and Caruana, 2013; Hinton et al., 2015) aims to train a smaller network (usually referred as a *student network*) with the supervision (or distillation of knowledge) from a larger network (referred as a *teacher network*). In KD, the student network is typically trained to match the prediction quality of the teacher network, and has been shown to reduce overfitting (Hinton et al., 2015; Lan et al., 2018). KD methods have been successfully used for various object detection tasks (Chen et al., 2017), including lesion segmentation on brain MR images (Lachinov et al., 2019; Hu et al., 2020; Vadacchino et al., 2021). The most commonly used distillation types include response-based (Hinton et al., 2015; Kim and Kim, 2017; Ding et al., 2019; Müller et al., 2019) and feature-based distillation (Romero et al., 2014; Zhou et al., 2018; Jin et al., 2019). In response-based distillation, the output logits from the softmax layer are softened (also known as *soft labels*) using a *temperature* parameter that acts as a regularization factor (Hinton et al., 2015). In the feature-based distillation, outputs of intermediate layers of the teacher model are used to train the student model (e.g., *hint learning* using outputs of hidden layers Romero et al., 2014; Jin et al., 2019 and parameter sharing of intermediate layers Zhou et al., 2018).

Based on the training methods, offline distillation (using a pretrained teacher model to train the student models) (Romero et al., 2014; Hinton et al., 2015), online distillation (training teacher and student models together) (Zhou et al., 2018; Guo et al., 2020) and self-distillation (where the student models from prior epochs become the teacher for the subsequent epochs) (Yang et al., 2019; Zhang et al., 2019) are most commonly used. Various techniques have also been proposed to improve the generalizability and the performance of the student models including using noisy data (Li et al., 2017; Sarfraz et al., 2019), adaptive regularization of distillation parameters (Ding et al., 2019) and adversarial perturbation of data for training (Xie et al., 2020). Multi-task learning methods have also been shown to provide good regularization, reducing the risk of over-fitting (Liu et al., 2019a; Ye et al., 2019). The auxiliary task could be a related task [e.g., auxiliary classification network in lesion segmentation (Yang et al., 2017)] or an adversarial task [e.g., adversarial training of domain predictor in domain adaptation networks (Ganin et al., 2016)].

So far, KD has never been used for CMB detection to the best of our knowledge. However, in this context, a teacher-student network could be highly beneficial. The teacher network is trained to differentiate CMBs from non-CMBs and then distil this knowledge for the student model to distinguish CMBs from various mimics. In this work, we, therefore, used for the first time a knowledge-distillation framework for accurate and fully automated detection of CMBs, given a single image modality. We propose a 3-step approach: in the first two steps we used 3D CNN models for CMB candidate detection and discrimination. In the third post-processing step, we used appearance-based attributes to reduce false positives. We tested our approach in the presence of mimics, across different datasets with different modalities and pathological conditions. Our main contributions are as follows:

- In the initial CMB candidate detection step (Section 2.2), we utilize the radial symmetry property of CMBs for more efficient candidate detection with high sensitivity.
- In the candidate discrimination step (Section 2.3), we use a knowledge distillation framework to create a light-weight student model from a multi-tasking teacher model, which overcomes the class imbalance between CMBs and the background, leading to the effective removal of false positives without reducing sensitivity.
- In the final post-processing step (Section 2.4), we exploit the structural properties of CMBs to further reduce false positives and improve precision by rejecting CMB mimics from the discrimination step.
- We evaluated our method on four different datasets (details are provided in Section 3). Through the experiments described in Section 4, we studied the contribution of the individual steps on the CMB detection performance, and also the effect of various modalities and different pathological conditions on the results. We also performed an indirect comparison of our results with existing methods at various stages of detection.

## 2. Materials and methods

In the following sections we describe the details of our method. We initially preprocess the input data and remove blood vessels in the images as specified in Section 2.1. The proposed method consists of three steps. (1) 3D CMB initial candidate detection (Section 2.2): this step takes in the preprocessed input images, performs fast radial symmetry transform (FRST), and applies a deep learning model on both input image and FRST output to generate an initial CMB candidate detection map. (2) CMB candidate discrimination (Section 2.3): the initial candidate detection map is taken as input and a knowledge distillation framework, involving multi-tasking teacher and student networks, is used on this input image as well as the FRST output, to discriminate between CMBs and non-CMB candidates (obtained from step 1). (3) Post-processing (Section 2.4): finally, the CMB discrimination map obtained from step 2 is fed into a post-processing step, which uses anatomical constraints to further reduce false positives from the discrimination map. Sections 2.5 and 2.6 provide information regarding the training and implementation details of the method.

### 2.1. Data pre-processing

We reoriented the T2*-GRE, SWI and QSM images to match the orientation of the standard MNI template, and skull-stripped the images using FSL BET (Smith, 2002). For T2*-GRE and SWI, we performed bias field correction using FSL FAST (Zhang et al., 2001). We also inverted the intensity values of the input volume by subtracting the intensity-normalized image (obtained by dividing intensity values by the maximum intensity) from 1, so that CMBs have higher intensities (a design choice to facilitate our choice of CNN layers—e.g., max-pooling layers). For QSM images, we only normalized the intensity values without inverting their intensity values since CMBs already appear hyperintense with respect to the background. We cropped the skull-stripped images closer to the brain edges to make the FOV tighter.

**Figure 1:**
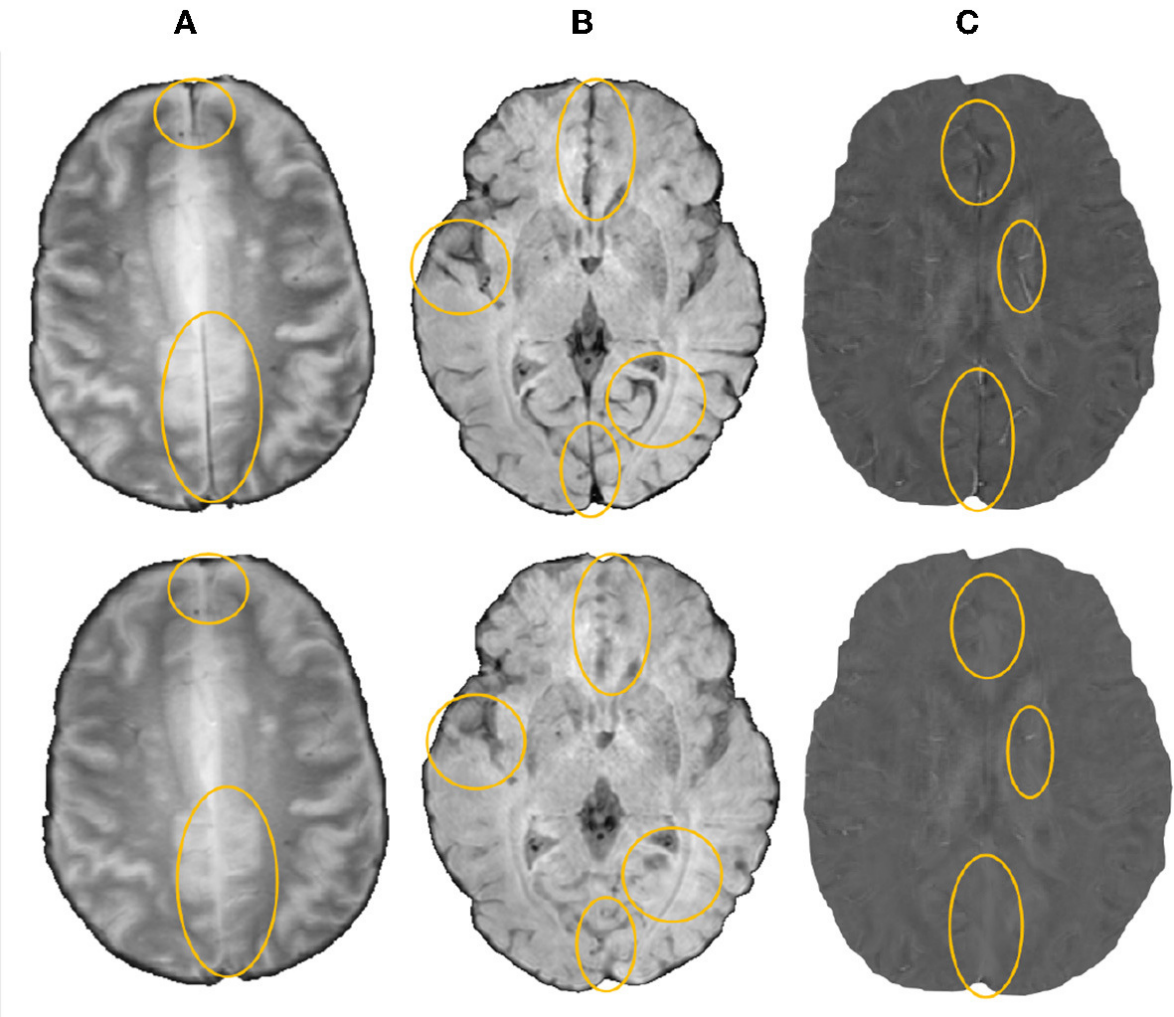
Sample images after removal of blood vessels and sulci (regions with major changes indicated by circles in the bottom row) shown for **(A)** T2*-GRE, **(B)** SWI, and **(C)** QSM modalities.

We then removed blood vessels, sulci and other elongated structures from the input image to reduce the appearances of CMB mimics using the method described in Sundaresan et al. (2022). Briefly, the method involves the extraction of edge and orientation-based features, using Frangi filters (Frangi et al., 1998) and eigenvalues of the structure tensor (Förstner, 1994), followed by *K*-means clustering to obtain the vessel masks. The masked regions were then inpainted using the mean of intensity values from the immediate non-masked neighboring voxels (within a 26-connected neighborhood). Figure 1 shows a few sample images (from various modalities) after the removal of vessels and sulci.

### 2.2. 3D CMB initial candidate detection

**Figure 2:**
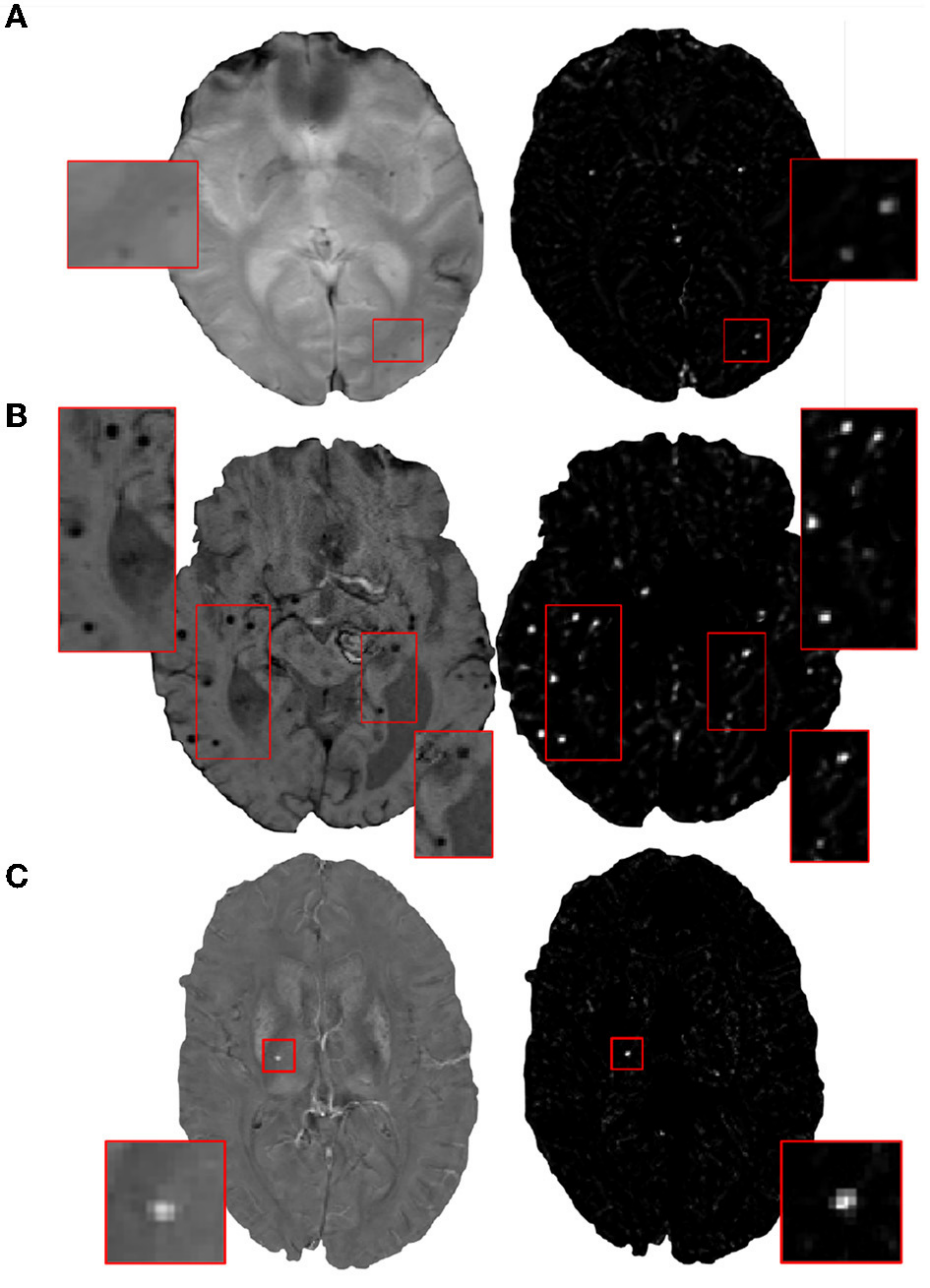
Examples of the final fast radial symmetry transform (FRST) outputs for various image modalities. The FRST outputs shown (in the right panel) for **(A)** T2*-GRE, **(B)** SWI, and **(C)** QSM images. Inset figures show the magnified versions of the regions indicated in the boxes.

In the initial candidate detection step, our main aim is to detect CMB candidates with maximum sensitivity, despite variations in their intensity characteristics and presence of mimics. The shape and size of CMBs are the main characteristics that could help differentiate them from mimics [e.g., flow voids, micro-hemorrhages, partial volume artefacts Greenberg et al. (2009)]. Since CMBs are circular, for the initial CMB candidate detection, in addition to the intensity characteristics, we also use the radial symmetry property of CMBs. We performed a fast radial symmetry transform (FRST) (Loy and Zelinsky, 2002) which uses a gradient-based operator to detect voxels with high radial symmetry. We calculated FRST at four radii (2, 3, 4, and 6 voxels) and then used their mean as the final FRST output (shown for different modalities in Figure 2).

During the training phase, for both the input modality and the FRST output, we split the 3D volumes into patches of size 48 × 48 × 48 voxels and provided them as two input channels to the 3D patch-based encoder-decoder model for initial candidate detection. We selected the patch-size of 48 voxels empirically—at this scale, the patches were large enough to overcome the effect of local noise and assign higher probabilities to CMB-like regions on experimented datasets described in Section 3. Note that we use patches only for training. During testing, we apply the trained model on whole 3D images.

**Figure 3:**
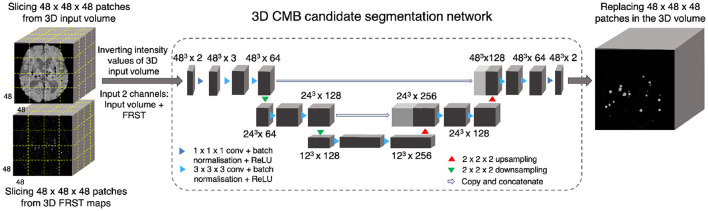
CMB initial candidate detection step. Block diagram of the candidate detection step with the architecture of the 3D encoder-decoder network based on shallow U-Net.

Figure 3 shows the block diagram of this initial candidate detection step and the architecture of the 3D encoder-decoder model. The architecture of the 3D encoder-decoder network at a scale *N* is based on a shallow U-Net. We trimmed the U-Net to a shallow architecture with two pooling layers. Since CMBs are small and sparse, the model is required to detect low-level features in limited-size patches, rather than global features generally learnt for larger lesions (e.g., stroke lesions) at the image-level. The choice of a shallow architecture is also in line with prior literature supporting their use when data is scarce (Amiri et al., 2019; Du et al., 2020). The input channels are converted into three channels by the initial 1 × 1 × 1 projection layer, followed by 3 × 3 × 3 convolution to get the initial filter channel depth of 64. The architecture consists of two consecutive 3 × 3 × 3 convolutional layers followed by the 2 × 2 × 2 max-pooling layer (in the encoder) or 2 × 2 × 2 upsampling layer (in the decoder). We added a 1 × 1 × 1 convolutional layer before the final softmax layer for predicting the probability maps *P*_*Cdet*_.

We used a combination of cross-entropy (CE) and Dice loss functions as the total loss function. In the CE loss function, we upweight the CMB voxels 10 times compared to the non-CMB voxels during training to compensate for the imbalance in the classes. Dice loss is based on the voxel-wise Dice similarity measure and aids in the accurate detection of edges and small CMBs in the patches.

### 2.3. CMB candidate discrimination

**Figure 4:**
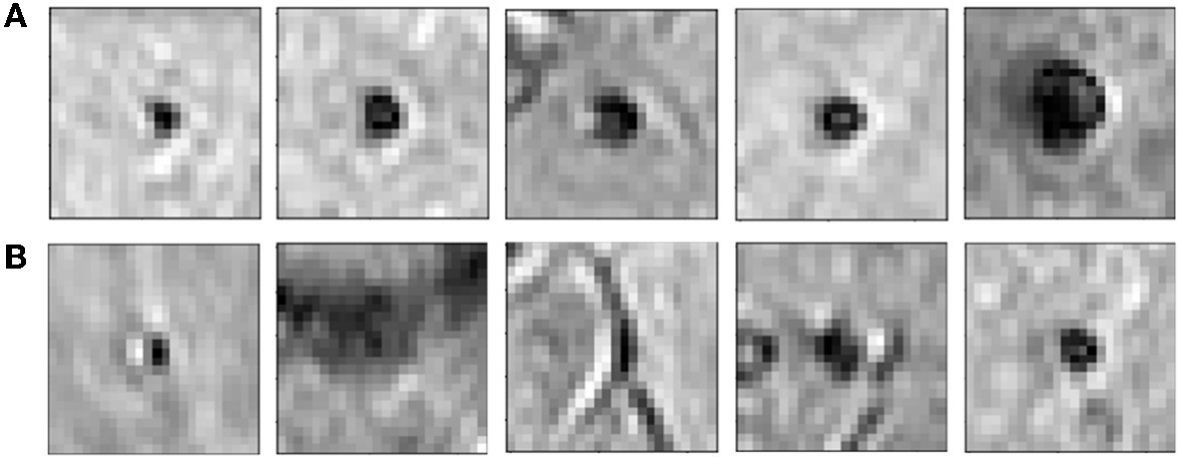
Examples of initial candidates detected in the first step. **(A)** CMB and **(B)** non-CMB patches are shown separately. Note that in most of the cases, non-CMB instances are quite similar to CMBs.

**Figure 5:**
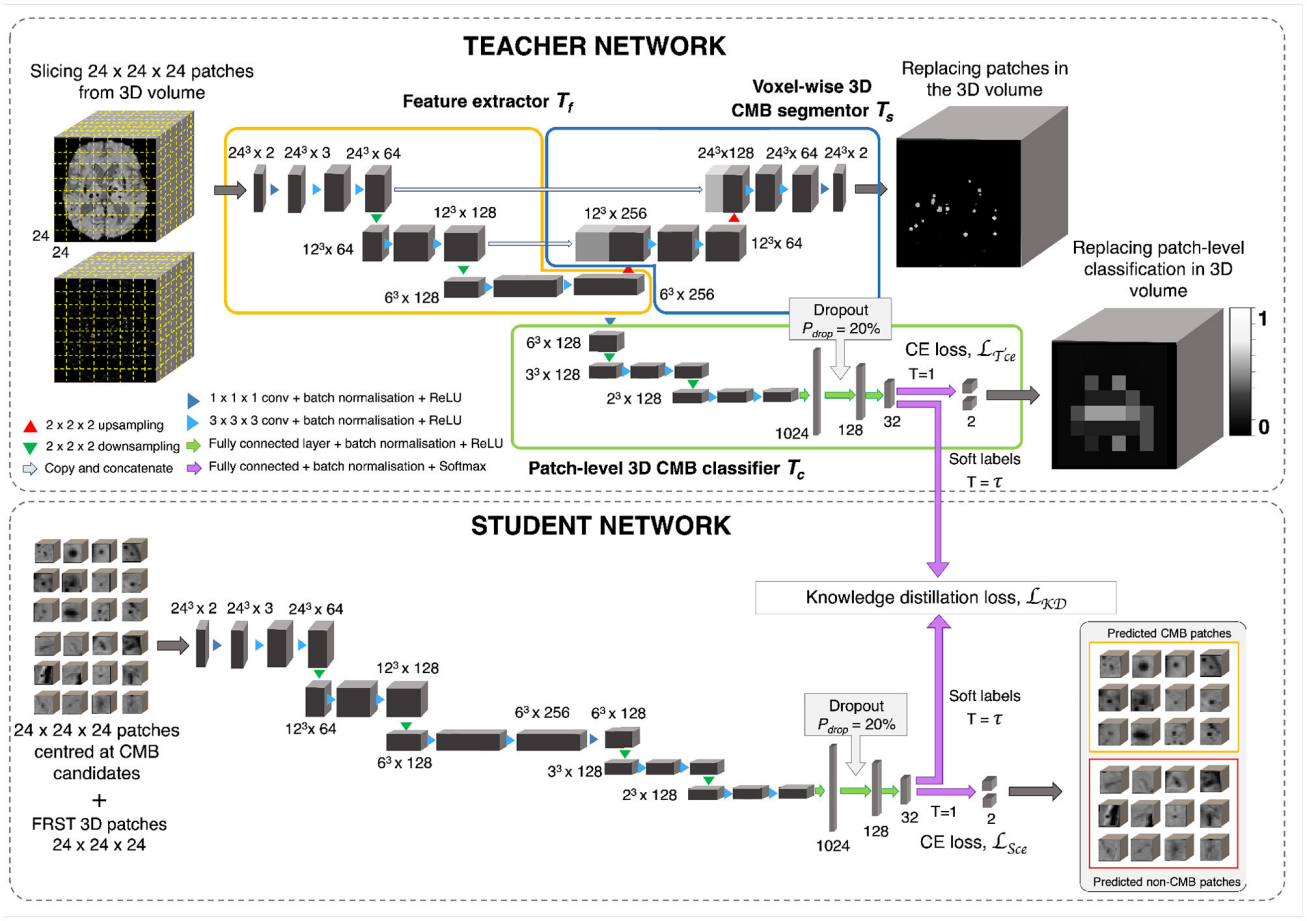
CMB candidate discrimination using knowledge distillation. **Top** panel shows the multi-tasking teacher model consisting of feature extractor *T*_*f*_ (orange), voxel-wise 3D CMB segmentor *T*_*s*_ (blue) and patch-level 3D CMB classifier *T*_*c*_ (green). The **bottom** panel shows the student model *S* for classification of CMB and non-CMB patches using the distillation of knowledge from the teacher model using the distillation loss *L*_*KD*_(*T*_*c*_, *S*).

The candidate discrimination step is more challenging than the initial candidate detection step, since the discrimination step needs to learn the subtle features to detect CMBs and discriminate them from other CMB mimics. To illustrate the complexity of the problem, Figure 4 shows instances of CMB and non-CMB patches that were all identified as CMB candidates in the initial detection step. In this step, we use a student-teacher framework for classifying true CMB candidates from FPs. We use two networks: (1) a teacher network that has a multi-tasking architecture and learns the task-based characteristics (in our case, CMB-related features) from a larger dataset of patches extracted in a sliding manner from the image; (2) a student model that has comparatively simpler architecture and is trained directly on more contextual patches centered at candidates detected from the initial candidate detection step (Section 2.2). For training the teacher model (and candidate detection stage), non-overlapping patches are used. They provide sufficient sample CMB candidates to achieve the main aim at this stage, which is to train the model with examples of CMBs in a more comprehensive manner (especially with a multitasking teacher model performing both classification and segmentation tasks). We aim to improve the classification accuracy of the student model, by guiding its training by using the information from the teacher model with response-based knowledge distillation. For both candidate detection and discrimination stages, patches were extracted only for training, whereas the trained models were applied on whole 3D images during testing. Figure 5 shows the proposed overall architecture, while the details of the student-teacher architecture and training are provided in the sections below.

#### 2.3.1. Teacher network with multi-task training

The teacher model uses a multi-tasking architecture consisting of three parts (1) feature extractor (*T*_*f*_), (2) voxel-wise CMB segmentor (*T*_*s*_) and (3) patch-level CMB classifier (*T*_*c*_). For the multi-tasking teacher model, we took the pretrained weights from the initial CMB candidate detection for *T*_*s*_ and added a patch-level CMB classifier arm (*T*_*c*_) using truncated normal variables for weight initialization (refer to Section 2.6). This helps to train the teacher model with more relevant initial weights for *T*_*s*_, since pretrained weights for *T*_*s*_ were already available from the candidate detection step. However, note that the teacher model can also be trained with random initialization for both *T*_*s*_ and *T*_*c*_. While the architecture of feature extractor + segmentor is the same as that of the model used in the initial candidate detection stage, the classifier arm consists of a projection layer with 1 × 1 × 1 kernel, followed by two consecutive 3 × 3 × 3 convolutional layers followed by a pooling layer in each level of abstraction. The output of the third layer of the encoder is fed into dense fully connected layers (FC). Three fully connected layers (FC-1024, FC-128, and FC-32) with 1,024, 128, and 32 nodes are then followed by a softmax layer. We added a dropout layer with a *drop probability* of 20% before the FC-128 layer. We extracted 24 × 24 × 24 adjacent patches from the input modality and FRST images, and provide them as 2-channeled inputs for training this model. While we used a patch-size of 48 for the detection stage, we used a smaller patch-size of 24 for this stage. This is because our main aim was to adapt the segmentor arm *T*_*s*_, initialized with pretrained weights (trained on patch-size of 48), to learn the lesion-level characteristics of initial CMB candidates from the local neighborhood obtained using smaller patches. The *T*_*f*_, made of a series of convolutional layers, extracts features that are helpful for both *T*_*s*_ and *T*_*c*_. Therefore, both *T*_*s*_ and *T*_*c*_ learn to improve the CMB segmentation and classification in a progressive manner since both are trained simultaneously with shared weights in *T*_*f*_. This means that *T*_*s*_ assigns high probability values to the CMB voxels in the CMB patches, while reducing the probability values of CMB-like mimics on the non-CMB patches. At the same time, *T*_*c*_ detects the patches with more CMB-like features (regions that are assigned higher probabilities by *T*_*s*_) as CMB patches with higher confidence and vice versa. In addition to the loss function to train *T*_*s*_ (specified in Section 3), we used a binary cross-entropy loss function for *T*_*c*_.

#### 2.3.2. Knowledge distillation using student network

The student model consists of a feature extractor and a patch-level classifier parts (*T*_*f*_ + *T*_*c*_), as the teacher model. However, while we provided non-overlapping, adjacent 24 × 24 × 24 patches for the teacher model, we extracted more meaningful input patches for the student model, centered at the detected initial CMB candidates for quicker learning. We trained the student model in an offline manner using response-based knowledge distillation (KD). For determining the centroids of the patches, we thresholded *P*_*Cdet*_ from the first step at a specific threshold *Th*_*Cdet*_ based on the performance values (for more details refer to Section 5.2). During testing, patches centered at candidates detected from the initial candidate detection steps are classified as CMB or non-CMB by the student model. Let the student model and teacher model classifier be *S* and *T*_*c*_, respectively. For the distillation of knowledge from the teacher model for training the student model, the loss function is given by


(1) $$\begin{array}{l} L=\alpha *L_{S}+\beta *L_{KD}\left(T_{c},S\right) \end{array}$$

where *L*_*s*_ is the student loss function, *L*_*KD*_(*T*_*c*_, *S*) is the KD loss and α, β are weighing parameters. We used the cross-entropy loss function as the student loss. For determining the KD loss, the targets are the class outputs predicted by the classifier of the teacher model (in the inference mode) on the same input as that of the student model. A temperature (τ) parameter is used in the softmax function to soften the target distribution. While τ = 1 provides the usual softmax outputs, higher values of τ soften the softmax outputs (as shown in Equation 2). The softmax function with τ is given by,


(2) $$\begin{array}{l} \sigma (z_{i},\;\tau )=\frac{exp(z_{i}/\tau )}{\sum_{j=1}^{N}exp(z_{j}/\tau )} \end{array}$$

where *N* is the number of classes. Compared to hard target distributions (closer to 0 or 1 for individual classes), softer target distributions (between 0 and 1) have been shown to aid in training a generalizable student model (Hinton et al., 2015), however, having very high τ might also be counter-productive in some cases. The optimal value of τ and the level of softness in the target distribution depends on specific applications, student/teacher network architectures and dataset characteristics. Temperature τ values between 2.5 and 4 have been shown to provide better results, while models with more units in the hidden layers may require higher τ values (Hinton et al., 2015). Using the temperature τ parameter, the KD loss is given by,


(3) $$\begin{array}{l} L_{KD}\left(T_{c},S\right)=KL\left(\sigma \left(z_{S},\;\tau \right),\sigma \left(z_{T_{c}},\;\tau \right)\right) \end{array}$$

where *KL* is the KL-divergence (distance between the class probability distributions of student and teacher classifier models). From Equation 1, the loss function is,


(4) $$\begin{array}{l} L=\alpha *CE\left(y_{S},\;\sigma \left(z_{S},\;\tau =1\right)\right)+\beta *KL\left(\sigma \left(z_{S},\;\tau \right),\sigma \left(z_{T_{c}},\;\tau \right)\right) \end{array}$$

where *y*_*S*_ are the target labels of the student model and *z*_*S*_ and *z*_*T*_*c*__ are the logits (inputs to the softmax layer) of the student and teacher classifier model. respectively.

### 2.4. Post-processing

**Figure 6:**
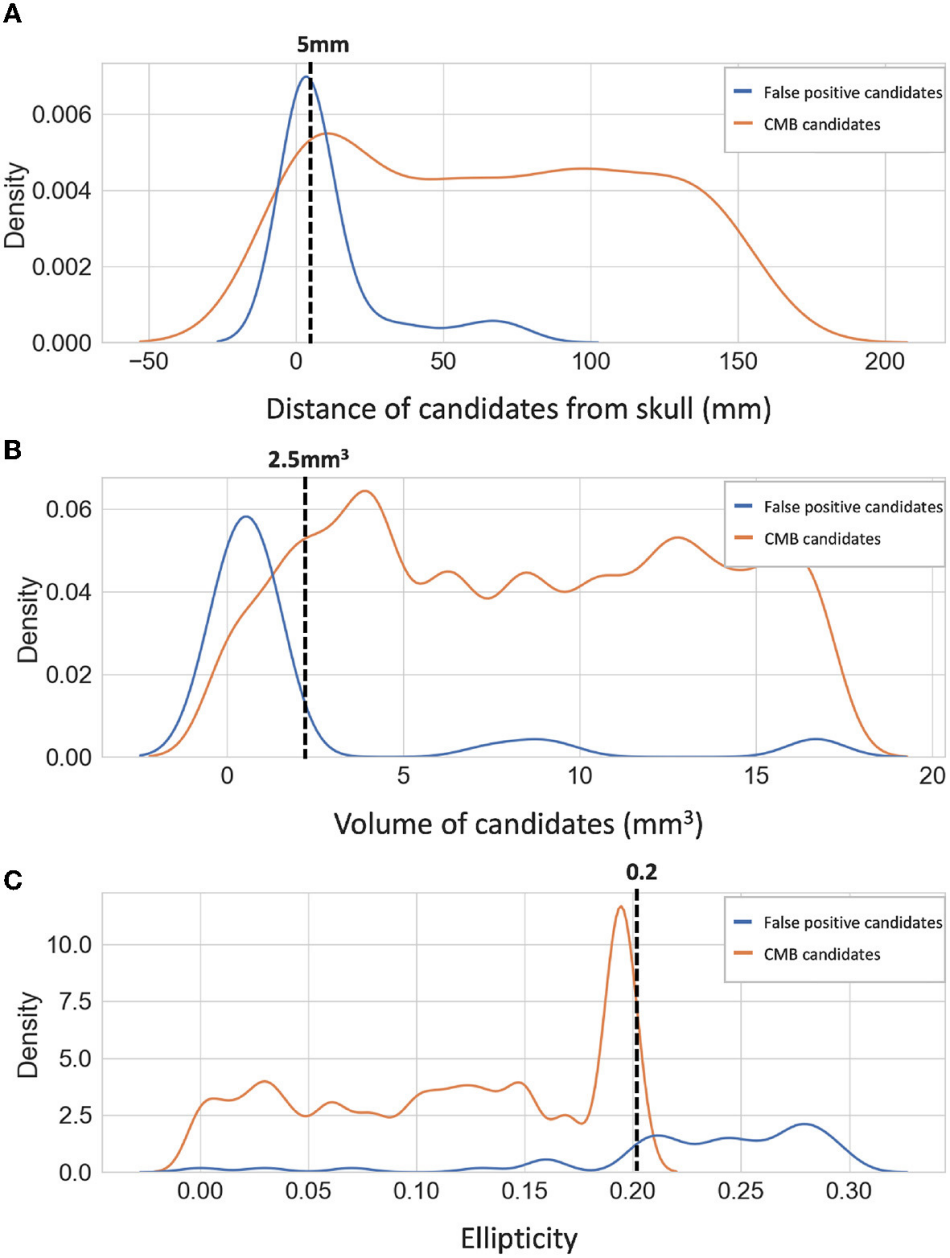
Density plots of candidate-level attributes. Density plots of **(A**) distance from the skull (mm), **(B)** volume (mm^3^), and **(C**) ellipticity of true CMB candidates (orange) and false positive candidates (blue) on an independent dataset (see Section 2.6). The filtering criteria are marked on the density plots with dotted lines.

We applied a threshold *Th*_*Cdisc*_ on the probabilities to discriminate CMB and non-CMB candidates. We set *Th*_*Cdisc*_ values empirically based on the performance metric values (refer to Section 5.2). Additionally, we removed the noisy stray voxels by filtering out the candidates with volume < 2.5mm^3^, removed the tubular structures (e.g., fragments of sulci near the skull) by filtering out candidates having higher ellipticity (>0.2) and removed the CMB candidates that are closer to the skull (< 5 mm from the brain mask boundary) to reject the FPs due to the sulci in the brain. The density plots for the above attributes for false positive and true CMB candidates on an independent dataset (that was used for hyperparameter tuning as specified in Section 2.6) are shown along with the cut-off criteria values in Figure 6. Note that this dataset was not later used for training or testing in the evaluation. The cut-off criteria values were determined empirically based on the attribute values as shown in density plots.

### 2.5. Data augmentation

Due to the small size of CMBs, transformations such as rotation and down-scaling could result in the loss of CMBs in the augmented data patches. Hence, we chose our data augmentation carefully, to inject variations in the data with minimal interpolation of intensity values. For the initial candidate detection step, we performed augmentation on the patches, increasing the dataset size by a factor of 10, using random combinations of the following transformations: translation, random noise injection and Gaussian filtering (with a small σ value). The parameters for the above transformations were chosen randomly from the ranges as specified as follows: (1) Translation: *x*-offset: [−15, 15], *y*-offset: [−15, 15] voxels, (2) Random noise injection: Distribution - Gaussian, μ = 0, σ^2^ = [0.01, 0.04], (3) Gaussian filtering: σ = [0.1, 0.2] voxels. We used similar augmentation for the discrimination step, increasing training data by a factor of 5.

### 2.6. Implementation details

For both candidate detection and discrimination steps, patches were extracted only for training. During the testing phase, the trained models were applied to the whole 3D images. For both CMB candidate detection and discrimination steps, we trained the networks using the Adam Optimiser (Kingma and Ba, 2014) with an epsilon (ϵ) value of 1 × 10^−4^. We used a batch size of 8, with an initial learning rate of 1 × 10^−3^ and reducing it by a factor 1 × 10^−1^ every 2 epochs, until it reaches 1 × 10^−6^, after which we maintain the fixed learning rate value. For both candidate detection and candidate discrimination training, we also empirically set the total number of epochs to 100 and used a criterion based on a patience value (number of epochs to wait for progress on validation set) of 20 epochs to determine model convergence for early stopping. We used the truncated normal variable (with σ = 0.05) for weights initialization and the biases were initialized as constants with a value of 0.1. For the student model in the discrimination step, we used a temperature τ = 4, α = 0.4, and β = 0.6, determined empirically using a trial-and-error method. We used a subset of a publicly available dataset (https://valdo.grand-challenge.org/Data/) consisting of a random sample of 20 subjects for hyper-parameter tuning and the empirical determination of parameter values in the post-processing step and loss functions. The initial candidate detection model took ~15 min per epoch for training and converged in around 80 epochs. For the candidate discrimination step, the teacher model took ~20 min per epoch for training and converged in around 80 epochs (this model was trained only once and was always used in inference mode for training student model on various datasets during cross-validation). The student model took only < 5 min per epoch and converged in ~50 epochs, thereby effectively reducing the training time for various datasets. We implemented the networks on NVIDIA Tesla V100 GPU, in Python 3.6 using Pytorch 1.2.0.

## 3. Datasets used

We used four datasets for the evaluation of our proposed method. The datasets consist of images from different modalities, that were acquired using different scanners with variations in acquisition protocols and from subjects with different pathological conditions and demographic characteristics. Histograms of subject-level CMB counts for the individual datasets (along with zoomed-in histograms for subjects with CMB count < 10) are shown in Supplementary Figure S1.

### 3.1. The UK Biobank (UKBB) dataset

From 14,521 subjects with usable imaging and non-imaging variables of interest (out of ~20,000 subjects from the January 2018 release of UKBB), we preselected 78 CMB candidate subjects using the method proposed in Sundaresan et al. (2022). Manual segmentations in the form of coordinates were annotated on SWI images for these 78 subjects by a trained radiologist (A.G.M). From those coordinates, the ground truth segmentation for each CMB was obtained by a region-growing-based method that in addition to a voxel's intensity also takes into account its distance from the seed voxel, and is constrained by a maximum radius of five voxels in-plane and three voxels through-plane. The age range of subjects is 50.8–74.8 years, mean age 59.9 ± 7.2 years, median age 57.8 years, female to male ratio F:M = 37:41. For SWI, 3D multi-echo GRE images were acquired using 3T Siemens Magnetom Skyra scanner with TR/TE = 27/9.4/20 ms, flip angle 15^*o*^, voxel resolution of 0.8 × 0.8 × 3 mm, with image dimension of 256 × 288 × 48 voxels. The QSM images were generated using a multi-step post-processing of phase data as described in Wang et al. (2021). Briefly, the method involved a combination of phase data of individual channels, phase unwrapping, background field removal, followed by dipole inversion. Total number of CMBs in this dataset: 186, mean: 2.4 ± 7.0 CMBs/subject, median: 1 CMB/subject.

### 3.2. The Oxford Vascular Study (OXVASC) dataset

The dataset consists of T2*-GRE images from 74 participants from the OXVASC study (Rothwell et al., 2004), who had recently experienced a minor non-disabling stroke or transient ischemic attack. The 2D single-echo T2*-GRE images were acquired using 3T Siemens Verio scanner with GRAPPA factor = 2, TR/TE = 504/15 ms, flip angle 20^*o*^, voxel resolution of 0.9 × 0.8 × 5 mm, with image dimension of 640 × 640 × 25 voxels. Age range 39.6–91.2 years, mean age 69.8 ± 14.6 years, median age 67.3 years, female to male ratio F:M = 36:38. Out of 74 subjects, 36 subjects had CMBs, and manual segmentations, labeled using T2*-GRE images, were available for all 36 subjects. Total number of CMBs: 366, mean: 10.2 ± 33.3 CMBs/subject, median: 3 CMBs/subject.

### 3.3. The tranexamic acid for intraCerebral hemorrhage 2 (TICH2) trial MRI sub-study dataset

The dataset consists of a subset of the MRI data used in (Pszczolkowski et al., 2022) obtained as part of the TICH2 trial (Sprigg et al., 2018). The dataset consists of images with variations in image dimension, spatial resolution and MR acquisition parameters (details in Dineen et al., 2018). The dataset used in this work consists of 115 SWI from the subjects with spontaneous intracerebral hemorrhage (ICH). Age range 29–88 years, mean age 64.76 ± 15.5 years, median age 66.5 years, female to male ratio F:M = 24:26. Out of 115 subjects, 71 subjects had CMBs and manual segmentations for CMBs were available for all 71 subjects. Additionally, microbleed anatomical rating scale (MARS, Gregoire et al., 2009) values were provided for the CMB subjects. For evaluation purposes, we included in the manual segmentation maps used in all our experiments all CMBs that were labeled as either “*definite*” or “*possible*”. Total number of CMBs: 849, mean: 11.9 ± 22.0 CMBs/subject, median: 3 CMBs/subject.

### 3.4. The stroke dataset from Hong Kong (SHK)

Originally, the dataset used in Dou et al. (2016) consisted of 320 SWI images in total, out of which 126 are subjects with stroke (mean age: 67.4 ± 11.3) and 194 are from normal aging subjects (mean age: 71.2 ± 5.0). In this work, we used a subset of 20 subjects that were publicly available from this dataset. Manual annotations in the form of CMB coordinates were available along with the dataset. From coordinates, ground truth segmentations were obtained with the same method used for the UKBB data (refer to Section 3.1). Another rater independently provided the manual segmentations on SWI images on the dataset, and we considered the union of both manual masks as our final ground truth. Total number of CMBs: 126, mean: 6.3 ± 8.8 CMBs/subject, median: 3 CMBs/subject.

## 4. Experiments

### 4.1. Performance evaluation metrics

We evaluated the CMB detection results at the lesion-level using the following metrics for a total number of CMBs over the individual datasets, as done in the existing literature:

- **Cluster-level TPR:** the number of true positive clusters (i.e., CMBs) divided by the total number of true clusters as given by,

(5) $$\begin{array}{l} \text{cluster-wise TPR}=\frac{TP_{clus}}{\left(TP_{clus}+FN_{clus}\right)} \end{array}$$

where *TP*_*clus*_ and *FN*_*clus*_ are true positive (overlaps with a ground truth cluster by at least one voxel) and false negative clusters, respectively.

- **Average number of FPs per subject (FPavg):** for a given dataset *D*, FPavg is defined as the ratio of the total number of detected FP clusters (*FP*_*clus*_, has no overlap with a ground truth cluster) to the number of subjects (or images) in the dataset, as given by,

(6) $$\begin{array}{l} FPavg=\frac{\text{Total number of }FP_{clus}}{\text{Number of subjects in}D} \end{array}$$

- **Cluster-wise precision:** the number of true positive clusters divided by the total number of detected clusters as given by,

(7) $$\begin{array}{l} \text{cluster-wise precision}=\frac{TP_{clus}}{\left(TP_{clus}+FP_{clus}\right)} \end{array}$$

We used 26-connectivity to form the clusters. In general, for cluster-wise TPR and cluster-wise precision, the higher the values the better while for FPavg, lower values are better. We used TPR and FPavg values for plotting a free-response receiver operating characteristics (FROC) curve, which is a plot of cluster-wise TPR vs. the average number of false positives per image/subject.

### 4.2. Ablation study: effect of knowledge distillation on CMB detection within the UKBB dataset

In this study, we evaluate the effect of individual steps, including the teacher-student distillation framework and the post-processing step on the CMB detection performance (using metrics specified in Section 4.1) on the UKBB dataset (using a training-validation-test split of 44-10-24 subjects, with 40 CMBs in the test data). To this aim, we calculated the above performance evaluation metrics at the following stages: after the initial CMB candidate detection (i) without using FRST output, (ii) using FRST output as an additional input channel, after candidate discrimination (iii) using the teacher model *T*_*c*_ alone, (iv) a classification network trained without the teacher model (trained independently using only CE loss function *L*_*S*_), (v) with knowledge distillation using student-teacher training and (vi) after final post-processing. For the classification model in (iv), we used only the student model architecture and provided as inputs the patches centered at the detected initial CMB candidates. For the student-teacher architecture used in (v), we provided adjacent patches sampled in a sliding manner as inputs for the teacher model and patches centered at initial CMB candidates as inputs for the student model.

### 4.3. Cross-validation of CMB detection on T2*-GRE and SWI images within individual datasets

We performed 5-fold cross-validation separately on T2*-GRE images from the OXVASC dataset and SWI images from the UKBB dataset, and evaluated the cluster-wise performance using the metrics specified in Section 4.1 for the whole dataset across subjects. Note that in both cases, for the candidate discrimination step, we used the teacher model pretrained on the UKBB dataset (for response-based distillation), while only the student model was trained on the individual datasets. Also, for this cross-validation, we used the hyper-parameters that were determined separately using an independent dataset specified in Section 2.6.

### 4.4. Evaluation of the generalizability of the proposed method across different datasets

For this experiment, we trained the proposed method on SWI images from 78 subjects from the UKBB dataset. We chose UKBB as training data since it is a large, open-access epidemiological study. The manually annotated subset of UKBB dataset used in this work provided larger training data than the other datasets. Moreover, UKBB dataset consists of both SWI and QSM modalities, the former is commonly used for detecting CMBs and the latter is effective for removing false positives. We used the hyperparameters mentioned in Section 2.6 and evaluated the trained model on data from different domains (e.g., variations in intensity profiles, scanners and acquisition protocols and demographics), using performance metrics specified in Section 4.1, under the following three scenarios:

1. Evaluation on the same dataset with different modalities: training data—UKBB (SWI), test data: UKBB (QSM)
2. Evaluation on different datasets with the same modality: training data—UKBB (SWI), test data: TICH2 (SWI), SHK (SWI)
3. Evaluation on different datasets with different modalities: training data—UKBB (SWI), test data: OXVASC (T2*-GRE)

**1. Evaluation on the same dataset with different modalities:** We evaluated the effect of change in the modality only on CMB detection by applying the method, that was trained on SWI images from the UKBB dataset, to the QSM images (intensities non-inverted for QSM, hence CMBs appear brighter than the background, similar to the preprocessed SWI) from the same subjects from the UKBB dataset.

**2. Evaluation on different datasets with the same modality:** We evaluated our method on different test datasets to observe the effect of scanner-related and population-level pathological variations on the CMB detection. We applied our method trained on SWI images from the UKBB dataset to SWI images from 115 subjects with intra-cerebral hemorrhages from the TICH2 dataset and SWI images from 10 healthy controls and 10 subjects with stroke from the SHK dataset.

**3. Evaluation on different datasets with different modality:** We evaluated our method trained on SWI images from the UKBB dataset on the T2*-GRE images from 74 subjects from the OXVASC dataset. The OXVASC data is quite different from the UKBB data not only in terms of modality, but also in terms of resolution, scanner and demographic/pathological factors. Hence, this scenario would provide a better indication of the method's generalizability in real world clinical applications.

For the above experiments, for the CMB candidate detection and discrimination steps, we used the threshold values (*Th*_*Cdet*_ and *Th*_*Cdisc*_) determined during 5-fold cross-validation on the UKBB dataset.

### 4.5. Indirect comparison of our results with the existing literature

Finally, we performed an indirect comparison of our results from the UKBB and OXVASC datasets with those of existing CMB detection methods in the literature.

## 5. Results

### 5.1. Ablation study: effect of knowledge distillation on CMB detection within the UKBB dataset

**Figure 7:**
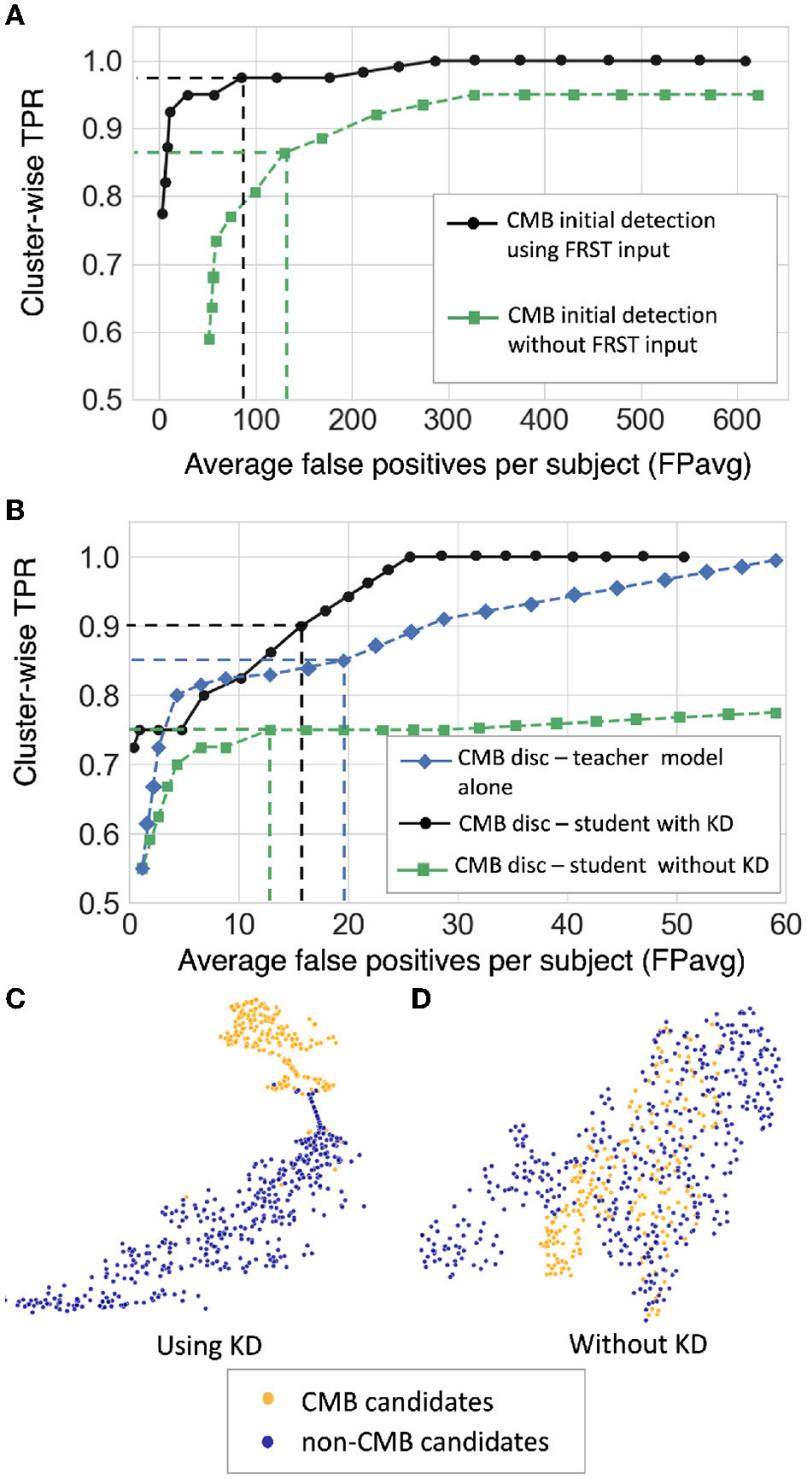
Results of the ablation study. **(A**) FROC curves at the CMB initial candidate detection stage using FRST output as an additional input channel (black solid •) and without using FRST output (green dashed ■), **(B)** FROC curves comparing the classification performance of the teacher model *T*_*c*_ (blue dashed ♦), the student model trained using KD from a teacher model (black solid •) and the same model trained independently without KD (green dashed ■). The horizontal and vertical dashed lines on the FROC curves indicate the best performance points determined at the knee-point of the curve with higher TPR (reported in Table 1) at *Th*_*Cdet*_ = 0.5 at the candidate detection step, *Th*_*Cdisc*_ = 0.29, 0.3, and 0.35 for the teacher model, models with and without KD, respectively in the candidate discrimination step. T-SNE plots showing feature embeddings at the FC-32 layer for CMB (orange) and non-CMB (dark blue) cases for **(C**) the student model trained using KD and **(D)** the model without using KD. In **(C)**, the plot shows better separability of features corresponding to CMB and non-CMB classes, indicating the ability of the model to discriminate well between these two classes.

**Figure 8:**
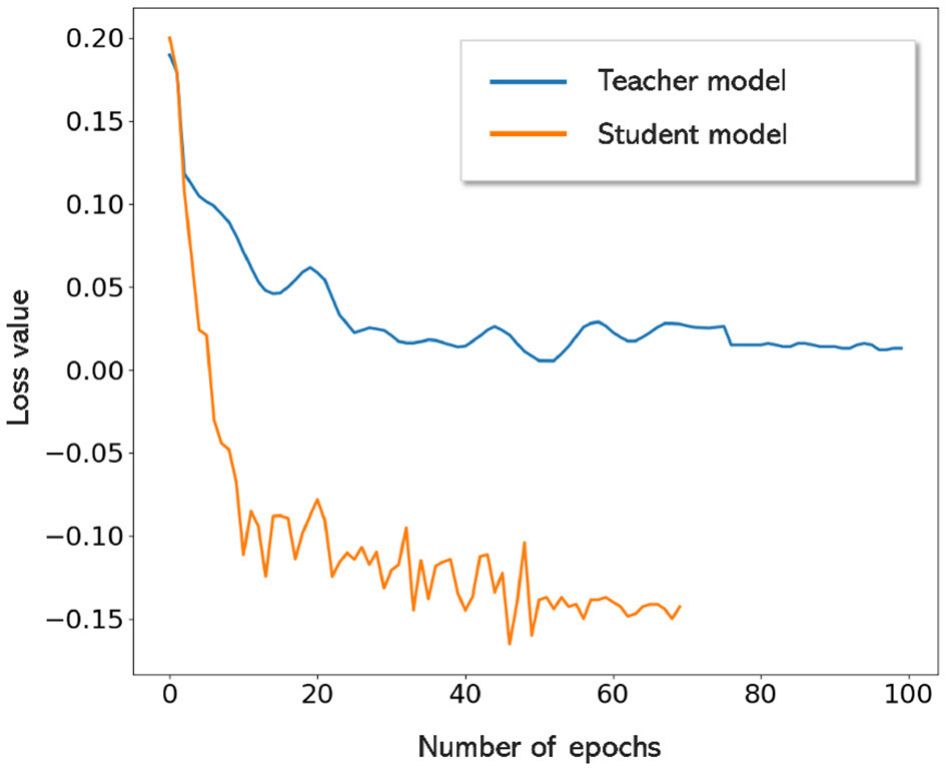
Convergence of teacher and student models. Student model trained with KD converges quicker and to lower loss values when compared to the teacher model.

Figure 7 shows the FROC curves for the initial CMB candidate detection and candidate discrimination steps of our method on the UKBB dataset. Table 1 reports the best performance points at the ‘knee-point' on the FROC curves for the first two steps, along with the performance metrics after the third step (post-processing). In the candidate detection step, the aim was to achieve higher cluster-wise TPR, to detect as many true CMBs as possible. Hence, the number of FPs was higher at this step (with the highest cluster-wise TPR of 0.975 at the *Th*_*Cdet*_ = 0.5), when compared to the subsequent steps. Using the FRST output as an additional input channel improved the sensitivity with slightly lower FPavg (Figure 7A). For the candidate discrimination step (Figure 7B), the FROC curves are shown for the comparison of the teacher model classification arm *T*_*c*_, student network trained with KD framework from the teacher model and classification network (with the same architecture as that of the student network) trained independently without KD from the teacher model. The performance at the candidate discrimination step is better with KD (cluster-wise TPR of 0.9 at *Th*_*Cdisc*_ = 0.3) than the model trained without KD (cluster-wise TPR of 0.75 at *Th*_*Cdisc*_ = 0.35), with the former showing an improvement of 0.02 in the cluster-wise precision (see Table 1). Also, the student model trained with KD performs better than the teacher model *T*_*c*_, with the improvement of 0.02 in cluster-wise precision. Moreover, the student model trained with KD converges quicker and to a much lower loss value when compared to the teacher model as shown in Figure 8.

**Table 1:** Ablation study: performance metrics after candidate detection, discrimination, and post-processing steps.

**Steps**	**Cl. TPR**	**FPavg**	**Cl. prec**
(i) Cand. det. without FRST	0.86	129.3	0.02
(ii) Cand. det. using FRST	0.975	85.3	0.03
(iii) Cand. disc. using teacher model *T*_*c*_	0.85	19.6	0.09
(iv) Cand. disc. without KD	0.75	12.8	0.09
(v) Cand. disc. using K	0.9	14.7	0.11
(vi) After postproc.	0.83	0.5	0.74

Cl. TPR and Cl. prec indicate cluster-wise TPR and cluster-wise precision, respectively. FPavg values at a fixed cluster-wise TPR value of 95% are provided in the Supplementary Table S3.

The t-stochastic neighbor embedding (t-SNE) plots show the separability between the features of the CMB and non-CMB classes as a measure of the model's ability to discriminate the classes from the learnt feature embeddings. For instance, the more separable the two classes are, the better the ability of the student model to discriminate between CMB class and mimics. Figures 7C, D show the feature embeddings of the last fully connected layer (FC-32) in the classification network (in the CMB discrimination step) for the CMB and non-CMB cases, trained with and without KD. The feature embeddings for the student model using KD were quite separable between the CMB and non-CMB cases, indicating the capability of the student model to learn the subtle differences in the features between CMB and non-CMB classes, using the information from the multi-tasking teacher model. The classification model without KD, on the other hand, showed substantially more overlap between the feature embeddings. The post-processing step improves the cluster-wise precision. Upon visual inspection, the main reductions in FPs were near the skull (e.g., sulci), penetrating blood vessels and stray noisy voxels. Regarding the contribution of individual attributes (e.g., shape, area and proximity to the skull) in FP reduction, we observed around 65, 25, and 15% of FP reduction after applying thresholds on distance of candidates from the skull, area and shape of candidates successively. Since the individual thresholds were determined as a part of hyper-parameter tuning on an independent dataset (Section 2.4) and the interaction between the three attributes' thresholds on FPavg is difficult to visualize, a separate FROC curve for the post-processing step is not shown. The majority of FPs rejected at this stage consists of candidates closer to the skull - these candidates passed the discrimination step since most of the CMBs in the training data (for the student model) were lobar CMBs and were closer to the skull. Hence the discrimination step (despite removing a large number of FPs near sulci) allows false predictions in this region. Having said that, it is worth noting that, in the post-processing step, a few true CMBs closer to the skull were also rejected as FPs, hence leading to a slight decrease in the cluster-wise TPR values.

### 5.2. Cross-validation of CMB detection on T2*-GRE and SWI images within individual datasets

**Figure 9:**
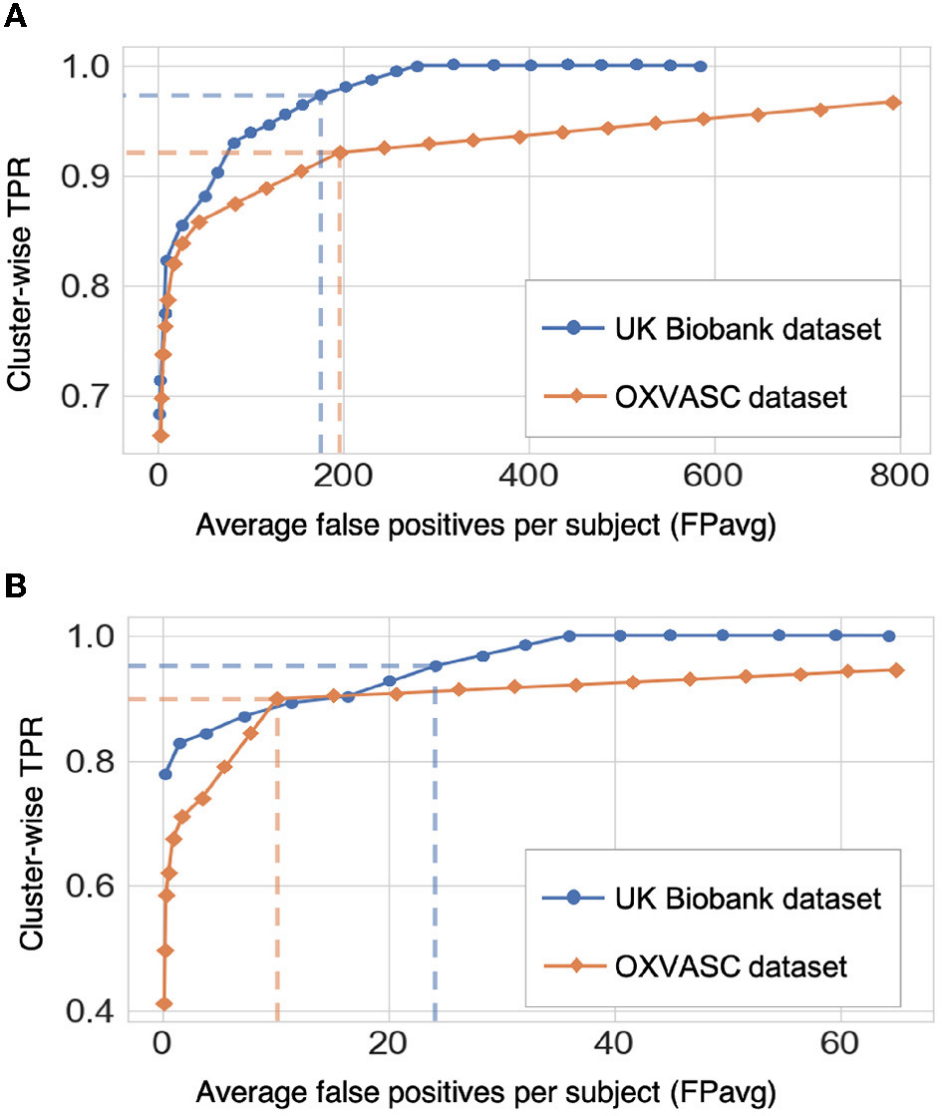
Results of the 5-fold cross-validation. FROC curves at **(A)** the CMB initial candidate detection stage and **(B)** the candidate discrimination stage on the UKBB (blue) and OXVASC (orange) datasets. The dashed lines on the FROC curves indicate the best performance points (reported in Table 2) at specific threshold points (threshold = 0.3 and 0.2 for the UKBB and OXVASC datasets, respectively for both candidate detection and candidate discrimination steps).

Figure 9 shows the FROC curves for CMB candidate detection and candidate discrimination steps of 5-fold cross-validation on whole images across all subjects on the UKBB and OXVASC datasets. Table 2 reports the best performance metrics at different steps of the cross-validation on the UKBB and OXVASC datasets. The proposed method achieved cluster-wise TPR values of 0.93 and 0.90 with FPavg of 1.5 and 0.9 at *Th*_*Cdet*_, *Th*_*Cdisc*_ = 0.3 and 0.2 on the UKBB and OXVASC datasets, respectively. The method provides higher cluster-wise TPR and FPavg values on SWI images (from the UKBB dataset) when compared to the T2*-GRE images from the OXVASC dataset. Even though the FPavg values were comparable at the candidate detection step for both datasets, the student model at the candidate discrimination step provided much lower FPavg on T2*-GRE images from the OXVASC dataset, thus providing a higher cluster-wise precision value. The FPavg values reduced substantially after the post-processing step with only a slight reduction in the cluster-wise TPR values.

**Table 2:** Cross-validation on the UKBB and OXVASC datasets: performance metrics at candidate detection, discrimination, and post-processing steps.

**Datasets**	**Steps**	**Cl. TPR**	**FPavg**	**Cl. prec**
UKBB (SWI)	C. det.	0.97	175.4	0.01
	C. disc.	0.95	24.2	0.09
	Postproc.	0.93	1.5	0.59
OXVASC (T2*-GRE)	C. det.	0.93	195.7	0.02
	C. disc.	0.91	10.1	0.29
	Postproc.	0.90	0.9	0.84

Cl. TPR and Cl. prec indicate cluster-wise TPR and cluster-wise precision, respectively. C. det - candidate detection, C. disc - candidate discrimination. FPavg values at a fixed cluster-wise TPR value of 95% are provided in the Supplementary Table S4.

**Figure 10:**
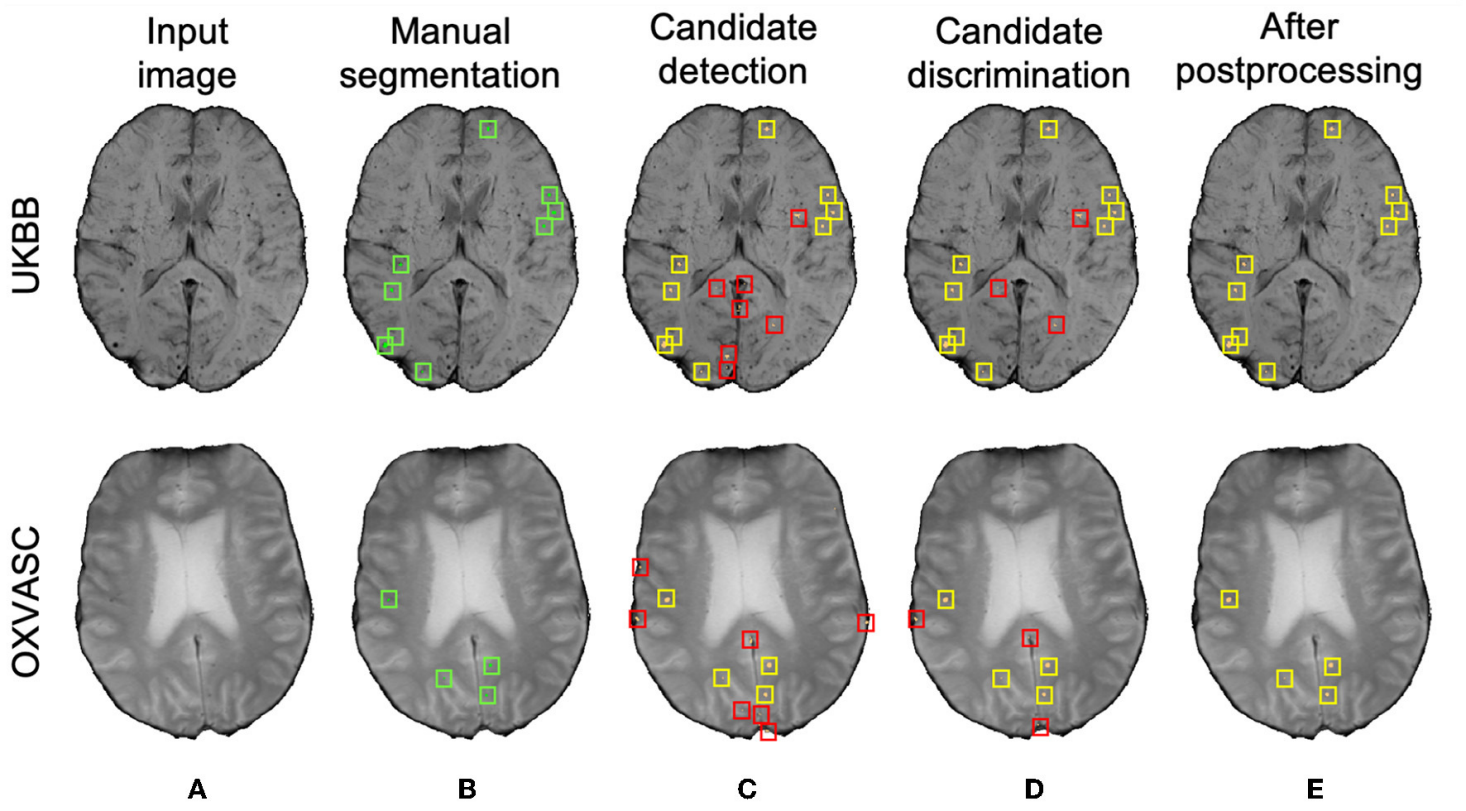
Sample cross-validation results on the UKBB (top panel) and the OXVASC (bottom panel) datasets. **(A)** Input image and **(B)** manual segmentations shown along with results at **(C)** CMB initial candidate detection, **(D**) candidate discrimination, and **(E)** after post-processing steps. True positive and false positive candidates are shown in yellow and red boxes, respectively for each step. Note that the displayed samples also include mimics (e.g., penetrating vessels and sulci) that appear similar to manually marked CMBs (in green boxes).

Figure 10 shows sample results of the cross-validation at various steps of CMB detection on the UKBB and the OXVASC datasets. In both UKBB and OXVASC datasets, the main sources of FPs in the initial candidate detection step are sulci, minor intensity/contrast variations in the brain tissue and small vessel fragments. While most of the penetrating blood vessels are segmented correctly as part of the background even at the candidate detection step (due to the vessel removal step, especially in the OXVASC dataset), the remaining FPs on/near the vessels are removed at the discrimination step. The post-processing step further reduced the stray noisy voxels and sulci regions closer to the skull, resulting in very few FPs on both datasets.

### 5.3. Evaluation of the generalizability of the proposed method across different datasets

**Figure 11:**
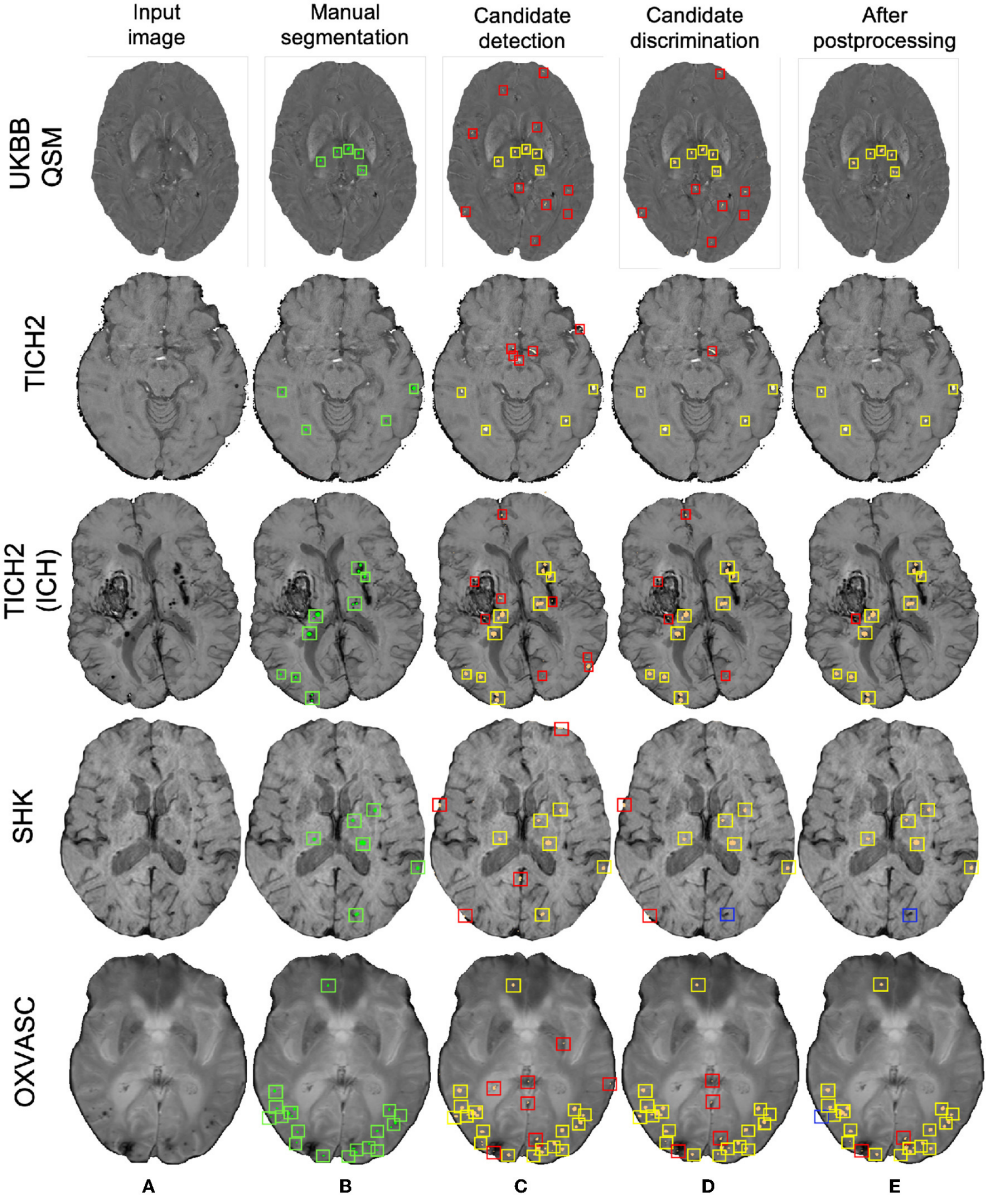
Sample results of the UKBB-trained method on the UKBB QSM, TICH2 (without and with ICH), SHK, and OXVASC datasets (from top to bottom panels). **(A**) Input image and **(B)** manual segmentations shown along with results at **(C)** CMB initial candidate detection, **(D)** candidate discrimination and **(E)** after post-processing steps. True positive, false positive and false negative candidates are shown in yellow, red, and blue boxes, respectively for each step. Note that the displayed samples also include mimics (e.g., penetrating vessels and sulci) that appear similar to manually marked CMBs (in green boxes).

Table 3 reports the performance metrics of the proposed method, when trained on the UKBB dataset and applied on the same dataset but different modality (UKBB QSM data), different datasets but the same modality (SWI from the TICH2 and SHK datasets) and different datasets and modality (T2*-GRE from the OXVASC dataset). We used *Th*_*Cdet*_ and *Th*_*Cdisc*_ values of 0.3 (determined from the cross-validation on the UKBB dataset) on the probability maps at the candidate detection step and on the patch-level probabilities at the discrimination step. Figure 11 shows sample results of the method, when applied on various datasets at various steps of CMB detection.

**Table 3:** Evaluation of the generalizability of the proposed method - trained on the UKBB SWI data and evaluated on the UKBB QSM, TICH2, SHK, and OXVASC datasets: performance metrics at candidate detection, discrimination and post-processing steps.

**Datasets**	**Steps**	**Cl. TPR**	**FPavg**	**Cl. prec**
UKBB (QSM)	C. det.	0.99	138.0	0.02
	C. disc.	0.91	40.3	0.04
	Postproc.	0.90	1.8	0.44
TICH2 (SWI)	C. det.	0.88	289.3	0.02
	C. disc.	0.83	42.8	0.10
	Postproc.	0.82	3.1	0.62
SHK (SWI)	C. det.	0.98	254.7	0.01
	C. disc.	0.94	43.6	0.09
	Postproc.	0.87	0.5	0.89
OXVASC (T2*-GRE)	C. det.	0.88	147.1	0.03
	C. disc.	0.85	53.7	0.07
	Postproc.	0.81	2.0	0.71

Cl. TPR and Cl. prec indicate cluster-wise TPR and cluster-wise precision, respectively. C. det, candidate detection; C. disc, candidate discrimination. FPavg at cluster-wise TPR value of 95% could not be provided since specific values of thresholds were applied.

Out of all datasets, the method achieved the highest cluster-wise TPR on the QSM dataset. On this dataset, the results were on par with the cross-validation results on the UKBB SWI data (with a slight decrease in the cluster-wise TPR and precision on QSM data). We obtained FPavg values of 1.8 FPs/subject—the FPs candidates were mainly due to minor susceptibility changes in the tissue and penetrating small blood vessels.

The method gave a cluster-wise TPR of 0.82 on the TICH2 dataset, despite the presence of ICH lesions (third row in Figure 11) in all subjects. The method provided the highest FPavg values in the initial candidate detection step (FPavg = 289.3 FPs/subject), possibly due to ICH edges and texture. However, the candidate discrimination step reduced the number of FPs and lowered the FPavg value to 42.8 FP/subject, which is comparable with other datasets. Even then, we obtained the highest FPavg after post-processing on this dataset with a cluster-wise precision of 0.62. Susceptibility artefacts at tissue interfaces and sulci were mainly detected as FPs in this dataset.

On the SHK dataset, while the first two steps (CMB detection and discrimination) provided consistently good cluster-wise TPR values (>0.90), the TPR value decreased at the post-processing step. Even then, on this dataset our method provided the lowest FPavg (0.5 FPs/subject) and the highest cluster-wise precision among all the datasets. The FPs in the candidate detection step were mostly regions of intersections of blood vessels closer to the sulci, especially the central sulcus.

On the OXVASC dataset, the method achieved the lowest cluster-wise TPR of 0.81. The high number of false negatives in this dataset, as suggested by the lower cluster-wise TPR, could be due to the reduced contrast between CMBs and normal brain tissue, unlike the SWI data used for training. Also, occasionally true CMB candidates quite close to the skull were removed in the post-processing step, having been mistaken as sulci.

### 5.4. Indirect comparison with existing methods

Table 4 provides a comparison of the proposed CMB detection method with existing fully automated methods. From the table, generally deep-learning-based methods performed better compared to conventional machine learning methods. Also, the methods using multiple modalities or using phase information in addition to SWI (Ghafaryasl et al., 2012; Liu et al., 2019b; Al-Masni et al., 2020; Rashid et al., 2021) showed better results. In fact, Al-Masni et al. (2020) showed that using phase in addition to SWI images improves the cluster-wise TPR by 5.6% (with only SWI: 91.6% and with SWI and phase: 97.2%) and Rashid et al. (2021) achieved the best CMB detection performance by using T2-weighted, SWI and QSM modalities. However, our proposed method uses a single modality (SWI or T2*-GRE), along with the FRST images (obtained from the input modality itself) and gives comparable results to state-of-the-art methods such as Liu et al. (2019b) and Al-Masni et al. (2020), and with lower FPavg compared to Dou et al. (2016). Also, our precision values on both UKBB and OXVASC datasets are better than existing methods including Bian et al. (2013), Fazlollahi et al. (2014, 2015), Dou et al. (2016). Even though not directly relevant to our work (since we performed all our evaluations at the image-level rather than at patch-level), we have reported an additional comparison of patch-level methods in Supplementary Table S5. While the methods using patch-wise evaluation (Chen et al., 2018; Lu et al., 2021b) provided good patch-level sensitivity and accuracies (shown in the Supplementary material), we cannot compare the performance of our method with those, since they used preselected CMB patches (from manually annotated CMB voxels) and comparable numbers of non-CMB patches as inputs. Also, the input CMB patches occasionally contained multiple CMBs, which makes the fair comparison with cluster-wise metrics highly difficult.

**Table 4:** Comparison of the performance of the proposed CMB detection method with existing conventional machine learning (ML) and deep learning (DL) methods.

**Methods**	**Datasets**		**Performance**		
	**Sequence(s) (# test subjects)**	**Total # CMBs**	**Cl. TPR**	**FPavg**	**Cl. Prec**
**ML methods**					
Bian et al. (2013)	SWI (10)	304	86.5%	44.9	
Fazlollahi et al. (2014)	SWI (41)	103	92%	FPavg_*CMB*_ - 6.7 FPavg_*nCMB*_ - 16.8	
Fazlollahi et al. (2015)	SWI (66)	231	87%	FPavg_*D*_ - 10.28, FPavg_*P*+*D*_ - 27.8	
Ghafaryasl et al. (2012)	T2*-GRE + PD (81)	183	91%	4.1	
Dou et al. (2015)	SWI (19)	161	80%	7.7	49%
Chesebro et al. (2021)	T2*-GRE, SWI (78)	64	95%	9.7 (SWI), 17.1 (T2*-GRE)	11% (SWI),7% (T2*-GRE)
**DL methods**					
Chen et al. (2015)	SWI (5)	55	89%	6.4	56%
Dou et al. (2016)	SWI (50)	117	93%	2.74	44%
Liu et al. (2019b)	Phase + SWI (41)	168	96%	1.8	(5-fold CV)
Al-Masni et al. (2020)	Phase + SWI (72)	188	94.3%	1.4	61.9%
Rashid et al. (2021) (Leave-one-out validation)	QSM + SWI + T2w (24)	~172	89%		49%
Proposed method	UKBB - SWI (78)	186	93%	1.5	59%
	OXVASC - T2*-GRE (74)	366	90%	0.9	84%

Cl.TPR, cluster-wise TPR; FPavg, average false positives per image/subject; Cl.Prec, cluster-wise precision; FPavg_*CMB*_, FPavg for CMB subjects; FPavg_*nCMB*_, FPavg for non-CMB subjects; FPavg_*D*_ - FPavg for “*definite*” CMB subjects; FPavg_*P*+*D*_, FPavg for “*definite and possible*” CMB subjects.

## 6. Discussion and conclusions

In this work, we proposed a fully automated, deep-learning-based, 3-step method for accurate lesion-level detection of CMBs on various datasets, irrespective of variations in population-level, scanner and acquisition characteristics. Our method uses a single input modality and the radial symmetry property of CMBs for the detection of CMB candidates with high sensitivity in the initial candidate detection step. For the candidate discrimination step, we trained a student classification network with the knowledge distilled from a multi-tasking teacher network for accurate classification of CMB candidates from non-CMB candidates. Our ablation study results show that the candidate discrimination and post-processing steps drastically reduce the number of FPs, and the use of the KD framework improves cluster-wise TPR values at the discrimination step. Our method achieved cluster-wise TPR values >90% with FPavg of < 1.6 FPs/subject during initial cross-validation on the UKBB and OXVASC datasets consisting of SWI and T2*-GRE images, respectively. On training the models on the UKBB dataset and applying them on different datasets with different demographic and scanner-related variations, the method showed a good generalizability across datasets, providing cluster-wise TPR values >80% on all datasets.

The initial vessel removal pre-processing step helped in reducing the number of FPs since blood vessels (especially the small ones closer to sulci) are one of the common mimics of CMBs. One of the main challenges in the vessel removal step is the potential removal of true CMBs that are very close to vessels. Hence, we removed only linear segments with low uniform width in this step. Therefore, this step removed the vessels and sulci that were more prominent and could lead to obvious FPs. This was especially effective for the SHK dataset, where the blood vessels and sulci had higher contrast and were distinctly different from CMBs (Figure 1B). While removing the linear, elongated structures from the images, we also aimed to leverage the radial symmetry property of CMBs. Toward that aim, using FRST maps, obtained from the input modality, as an additional input channel helped the candidate detection model in learning contextual features, leading to the detection of more true positive CMBs as shown in Figure 7A in the ablation study.

The main objective of the candidate detection step is to detect as many true positive candidates as possible, with a trade-off of high FPavg, because any of the CMB candidates missed in this stage cannot be recovered in the subsequent steps. From the ablation study, given high FPavg in the candidate detection step, the student model trained using KD reduced FPavg approximately by a factor of 4 with a smaller decrease in cluster-wise TPR, when compared to the classification model trained without KD. The advantage of the teacher model in the proposed discrimination step was 2-fold: (1) its ability to learn the contextual features that are salient for both voxel-wise CMB detection and patch-level CMB/non-CMB classification and (2) the use of a multi-tasking framework with *T*_*s*_ providing high cluster-wise TPR, while *T*_*c*_ providing a patch-level regularization by penalizing false positive detection, reducing the chance of over-fitting and false classifications. We observed that the multi-tasking framework, together with the upweighting of the CMB classes in the loss function, reduced the effect of class imbalance between CMB and non-CMB patches (note that the model trained without KD is slightly biased toward the non-CMB class, evident from the lower cluster-wise TPR and FPavg values in Table 1). The *T*_*c*_ component of the teacher model classifies CMB from non-CMB mimics, which enhances the capability of the student networks to differentiate CMBs from non-CMBs, evident from better cluster-wise TPR and precision for the student model with KD. This is also shown in t-SNE plots in Figures 7C, D, where the classes are more separable for the KD case. Additionally, we also provided the input patches centered on the detected initial CMB candidates to the student model. This, in addition to the distilled knowledge from the teacher, enabled the model to focus on the pattern at the center of the patches for accurate classification of CMB patches. This was especially useful to remove the fragments of blood vessels (e.g., intersections and branching points) missed in the vessel removal step as seen in Figures 10D, 11D. Regarding the parameters used in KD, using a higher temperature (τ) results in softened softmax values between classes and has been shown to typically provide the knowledge (also known as *dark knowledge*) for training a generalizable student network (Hinton et al., 2015). However, given the similarities in the characteristics of CMBs and mimics, having very high τ values could lead to misclassifications. Our main aim was to achieve a good hard prediction to differentiate the CMB class from the non-CMB class, while at the same time to transfer the knowledge from the teacher model to the student model. Hence we empirically chose an optimal τ value of 4 (that provided smoother softmax values without affecting the CMB/non-CMB prediction) based on manual tuning. Further removal of FPs in the post-processing resulted in the drastic improvement in the cluster-wise precision. The use of a post-processing step based on shape and spatial criteria has been shown to be beneficial in other studies (see Supplementary Table S2 for an overview). Given the big performance improvement after this step, we also tested the effect of applying post-processing at different stages of our proposed method. We found that, despite improving cluster-wise precision in all cases, the most beneficial effect was to use it as the final step of our 3-stage method (see Supplementary Table S1). Noise reduction or smoothing during pre-processing might lead to a loss of CMBs (even for data augmentation, very small σ values were chosen carefully). Therefore, small intensity and texture variations (mainly in the sub-cortical and lobar regions) led to the detection of FPs, which were removed in the post-processing step.

As for the cross-validation results within individual datasets, the method achieved the highest cluster-wise TPR values on SWI images (from the UKBB dataset), while providing the lowest FPavg and the highest cluster-wise precision on T2*-GRE images (from the OXVASC dataset). This could be due to the fact that CMBs appear with a higher contrast on SWI compared to T2*-GRE images due to the blooming effect. This also affects most of the CMB mimics as well, increasing their contrast on SWI, leading to high cluster-wise TPR but also high FPavg. Also, T2*-GRE images had a smoother texture when compared to SWI (Figure 10), resulting in less noisy FRST maps, hence leading to the improved performance metrics at the candidate discrimination step in the OXVASC dataset. However, the FPavg value at the initial candidate detection step was higher for the OXVASC dataset due to the lower voxel resolution in the *z*-direction (5 mm), leading to partial volume artefacts and making it highly difficult to differentiate between small sulci closer to the skull and CMBs.

On evaluating the generalizability of our method across various datasets, our method trained on the SWI data from the UKBB dataset showed good generalizability on QSM images from the same dataset, with comparable performance to the cross-validation results on the SWI data. Regarding the performance after individual steps, in the initial candidate detection step, the method provided the highest cluster-wise TPR values with the lowest FPavg values on the QSM data (even lower than with UKBB SWI data) since QSM shows a better separation of diamagnetic mimics from CMBs. However, due to local tissue susceptibility variations (which is quite different from the SWI training data), the FPavg in the candidate discrimination step was higher that it was when using the SWI data. Finally, the post-processing step effectively removed the stray voxels due to noisy susceptibility variations (that were extremely small and hence were below the 2.5 mm^3^ threshold) and reduced the FPavg value to 1.8 FPs/subject. It is worth noting that, since the same subjects were used for training (SWI data for training and QSM data for testing), the results are likely to be biased. That is, the model could have learnt the overall locations of CMBs for the training subjects, rather than the modality-invariant features. However, we believe that the use of patches, rather than whole slices or volumes, at both steps would reduce the chance of biased assessment.

For the datasets consisting of the same modality as that of the training data (SWI) but from different populations, the method was affected by the presence of additional pathological signs (e.g., ICH in the TICH2 dataset). In the TICH2 dataset, the noisy texture of the hemorrhage regions and their edges led to the highest FPavg value in the initial candidate detection step. In terms of FPs, we found that additional pathological signs, that were not the part of training, affected the method more than the modalities. For instance, among the OXVASC (different modality from the training SWI data) and TICH2 datasets (same modality), even though both are pathological datasets, the greater prevalence of confounding “CMB-like” signs in TICH2 resulted in higher FPavg in the TICH2 dataset. Among all the datasets we used, the SHK dataset had high contrast, low noise and a better than average resolution making vessels and sulci easy to remove in this dataset. Moreover, this dataset has the same modality as that of the training data, and hence both candidate detection and discrimination step models performed well (and cluster-wise TPR values comparable even with that on the UKBB SWI data). However, during the post-processing step, a few CMBs near the sulci, closer to the skull were misclassified as FPs resulting in lower cluster-wise TPR. The OXVASC dataset was quite different from the training SWI data and from other datasets, since it shows lower contrast between CMB and background as shown in Figure 11. Hence, providing FRST as the second input channel was particularly useful for this dataset, since the FRST relies more on the radial symmetry nature of CMBs at different radii (we used 2, 3, 4, and 6 as specified in Section 2.2) rather than its intensity contrast with respect to the background. Hence, on the OXVASC dataset the FRST maps had the same contrast as that of other modalities (as seen in Figure 2A) aiding in the detection of subtle CMBs. Since the estimation of FRST maps does not require any additional modality other than the input modality, our method effectively uses a single image modality and provided results comparable to existing methods that use multiple modalities (Ghafaryasl et al., 2012; Al-Masni et al., 2020).

Concluding, we proposed a fully automated method using deep learning for CMB candidate detection, and candidate discrimination with a knowledge distillation framework, followed by post-processing filtering using structural and anatomical properties. Our method achieved cluster-wise TPR values of >90% with FPavg < 1.6 FPs/subject on T2*-GRE and SWI modalities, on par with the state-of-the-art, and gave better precision than existing methods. When the models were trained on SWI data and applied on QSM images from the same dataset, the method achieved a cluster-wise TPR ~90%. On applying the trained method to other datasets consisting of data from different populations and acquired using different scanners and protocols, our method gave a cluster-wise TPR > 81%, despite the presence of other major pathologies. The Python implementation of the proposed method is currently available in https://github.com/v-sundaresan/microbleed-detection. The user guide (readme.md) provided via the above link provides more information regarding the scripts, input file formats and prediction times of the implementation. The tool yields high predictive performance on various modalities used in clinical settings. Also, given its short prediction time (< 5 min/scan), it has the potential to be used to assist clinicians by reducing the time taken for assessing individual scans, which would also benefit patients in the long run. Also, the CMB segmentation maps obtained from the tool could be used for obtaining an automated rating of CMBs (i.e., total CMB count, spatial distribution and size). One of the future directions of this research would be to improve the generalizability of the proposed method using various domain adaptation techniques, to overcome the effect of scanner- and population-related variations. Another clinically focused avenue of this research could be to develop automated algorithms to rate the CMBs based on their size and distribution, which would be useful in studying their clinical impact.

## Data availability statement

The datasets presented in this article are not readily available because Requests for data from the OXVASC Study will be considered by PR in line with data protection laws. The TICH-2 MRI sub-study data can be shared with bona fide researchers and research groups on written request to the sub-study PI RD (rob.dineen@nottingham.ac.uk). Proposals will be assessed by the PI (with advice from the TICH-2 trial Steering Committee if required) and a Data Transfer Agreement will be established before any data are shared. The UK Biobank and Hong Kong (HK) datasets are available to researchers through an open applications via https://www.ukbiobank.ac.uk/register-apply/ and http://www.cse.cuhk.edu.hk/~qdou/cmb-3dcnn/cmb-3dcnn.html, respectively. Requests to access the datasets should be directed to rob.dineen@nottingham.ac.uk; peter.rothwell@ndcn.ox.ac.uk.

## Ethics statement

OXVASC was approved by the South Central—Oxford A Research Ethics Committee (Research Ethics Committee reference number: 05/Q1604/70). Human subjects: UK Biobank has approval from the North West Multi-center Research Ethics Committee (MREC) to obtain and disseminate data and samples from the participants (http://www.ukbiobank.ac.uk/ethics/), and these ethical regulations cover the work in this study. Written informed consent was obtained from all participants. The TICH-2 trial obtained ethical approval from East Midlands (Nottingham 2) NHS Research Ethics Committee (Reference: 12/EM/0369) and the amendment to allow the TICH2 MRI sub-study was approved in April 2015 (amendment number SA02/15). The patients/participants provided their written informed consent to participate in this study.

## Author contributions

VS contributed to conceptualization, methodology, software, validation, formal analysis, investigation, visualization, and wrote the original draft. CA performed investigation, methodology, and software. GZ, AM, RD, PR, DA, and NS contributed to resources. AM and RD also contributed to data curation. DA contributed to data curation and investigation. CW, KM, BT, FA-A, and SS contributed to resources and data curation. LG contributed to conceptualization, methodology, data curation, and project administration. MJ contributed to conceptualization, methodology, funding acquisition, and project administration. GZ, MJ, and LG contributed to supervision. All authors contributed to manuscript revision, read, and approved the submitted version.
