# Supplementary material for: Automated detection of cerebral microbleeds on MR images using knowledge distillation framework
Source: Front Neuroinform. 2023 Jul 10;17:1204186. doi: 10.3389/fninf.2023.1204186 (PMC10363739; doi:10.3389/fninf.2023.1204186)
Supplement: Supplementary file 1 [file Data_Sheet_1.pdf]

# Automated Detection of Cerebral Microbleeds on MR images using Knowledge Distillation Framework - Supplementary Material

## 1 DATASETS USED: HISTOGRAMS OF SUBJECT-LEVEL CMB COUNTS

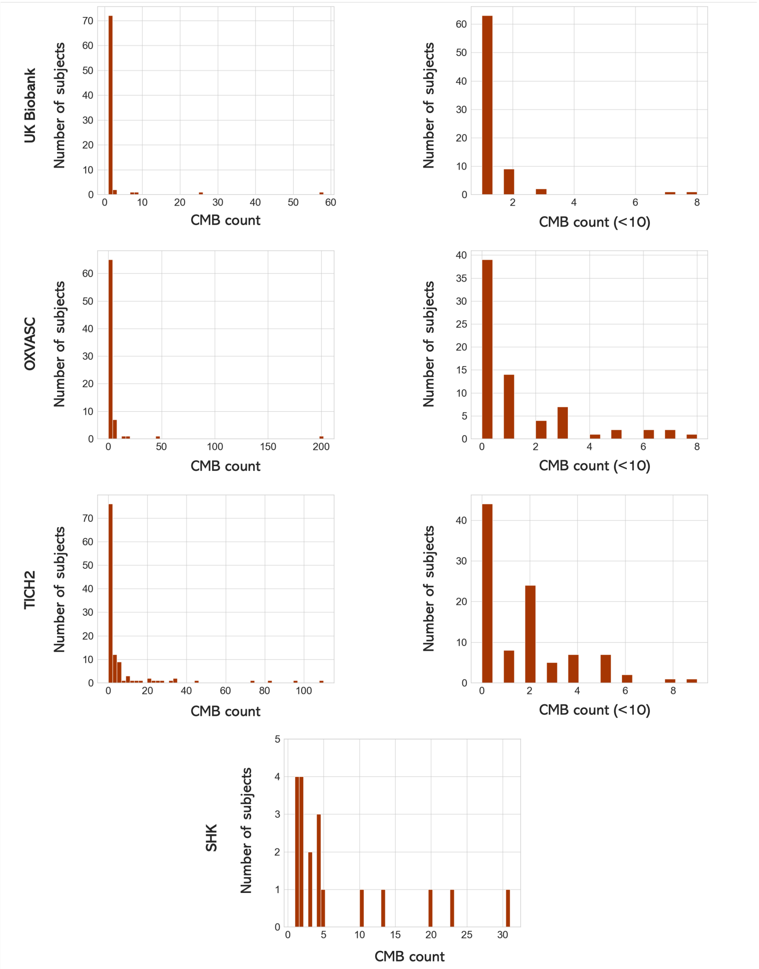


Figure S1: Histograms of the subject-level CMB counts based on manual segmentations for UKBB, OXVASC, TICH2 and SHK datasets. Since most of the subjects from UKBB, OXVASC and TICH2 have CMB counts $< 10$, zoomed-in histograms are shown for those subjects with CMB counts $< 10$ for the datasets.

## 2 EFFECT OF POSTPROCESSING: ABLATION STUDY AND COMPARISON OF EXISTING METHODS

In order to study the impact of postprocessing on individual steps, we performed additional experiments by applying postprocessing to the steps described in the ablation study. We considered 5 stages in our study:

- Case (i): Initial CMB candidate detection without using fast radial symmetry transform (FRST) output + postprocessing,
- Case (ii): nitial CMB candidate detection using FRST output as an additional input channel, followed by postprocessing,
- Case (iii): Candidate discrimination using the teacher model Tc alone + postprocessing,
- Case (iv): Candidate discrimination using a classification network trained without the teacher model (trained independently using only cross-entropy loss function LS) + postprocessing,
- Case (v): Candidate discrimination using with knowledge distillation using student-teacher training + postprocessing.

Please note that postprocessing is applied only at the end of each step. For instance, for (iii) – (v), we did not applying postprocessing at the candidate detection step, but rather at the end of candidate discrimination for fair comparison.

The results of our experiments is provided in Table S1. Note that case (v) of table S1 is the same as that of (vi) of Table 1 in the manuscript (since we applied postprocessing after Cand. disc. with KD in (vi) of table 1). As shown in the table, applying postprocessing in the individual stages improved their corresponding cluster-wise precision, however reduced their cluster-wise TPR values. Note that this reduction in the cluster-wise TPR in the initial candidate detection stage could lead to lower performance in the candidate discrimination stage.

**Table S1.** Ablation study: postprocessing (PP) applied to the candidate detection and discrimination steps. Cl. TPR and Cl. prec indicate cluster-wise TPR and cluster-wise precision respectively.

| Steps | Cl. TPR | FPavg | Cl. prec |
|---|---|---|---|
| (i) Cand. det. without FRST + PP | 0.78 | 110.6 | 0.18 |
| (ii) Cand. det. using FRST + PP | 0.90 | 69.8 | 0.29 |
| (iii) Cand. disc. using teacher model $T_c$ + PP | 0.75 | 7.1 | 0.15 |
| (iv) Cand. disc. without KD + PP | 0.68 | 3.5 | 0.27 |
| (v) Cand. disc. using KD + PP | 0.83 | 0.5 | 0.74 |

Our postprocessing step rejects false positives based based on attributes such as shape, volume and proximity to the skull. The majority of the existing state-of-the-art methods use a postprocessing step involving criteria based on attributes similar to those used in our method for reducing false positives. For instance, shape-based and volume-based criteria [3], feature-based classification [4,5], information from phase and QSM maps in multimodal methods [5], proximity to blood vessels and tissue boundaries [6] have been used for reducing false positives in existing methods. Table S2 shows the stage-wise performance of CMB detection reported in existing methods.

**Table S2.** The stage-wise performances reported for existing CMB detection methods. Sens - sensitivity, FPavg - Average false positives per image/ subject, Prec - precision, FPavgCMB - FPavg for CMB sub jects, FPavgnCMB - FPavg for non-CMB sub jects, FPavgD - FPavg for 'definite' CMB subjects, FPavgP+D - FPavg for 'definite and possible' CMB subjects.

| Methods | Datasets | | Performance | | |
|---|---|---|---|---|---|
| | **Sequence(s) (# test subjects)** | **Total # CMBs** | **CMB screening step** | **CMB discrimination step** | **FP reduction step** |
| ***ML methods*** | | | | | |
| Bian et al., [1] | SWI (10) | 304 | FPavg - 287.7 | | Sens - 0.865, FPavg - 44.9 |
| Fazlollahi et al., [2] | SWI (41) | 103 | Sens - 0.98, FPavg -695 | Sens - 0.92, $FPavg_{CMB}$ - 6.7 $FPavg_{nCMB}$ - 16.8 | |
| Fazlollahi et al., [3] | SWI (66) | 231 | Sens - 0.97, $FPavg_{D}$ - 669, $FPavg_{P+D}$ - 1373 | Sens - 0.87, $FPavg_{D}$ - 10.28, $FPavg_{P+D}$ - 27.8 | |
| Ghafaryasl et al., [4] | T2*-GRE + PD (81) | 183 | Sens - 0.98, FPavg -705 | Sens - 0.92, FPavg - 19 | Sens - 0.91, FPavg - 4.1 |
| Dou et al., [5] | SWI (19) | 161 | Sens - 1.00, FPavg - 807 | Sens - 0.94, FPavg - 190 | Sens - 0.8, FPavg - 7.7, Prec - 0.49 |
| ***DL methods*** | | | | | |
| Chen et al., [6] | SWI (5) | 55 | Sens - 0.96, FPavg $>$ 800 | Sens - 0.89, FPavg - 6.4, Prec - 0.56 | |
| Dou et al., [7] | SWI (50) | 117 | Sens - 0.98, FPavg -282 | Sens - 0.93, FPavg - 2.74, Prec - 0.44 | |
| Liu et al., [8] | Phase + SWI (41) | 168 | Sens - 0.99, FPavg -276.8 | | Sens - 0.96, FPavg -1.8 |

# 3 ABLATION STUDY: PERFORMANCE METRICS AT CLUSTER-WISE TPR OF 95%

**Table S3.** Ablation study: performance metrics after candidate detection, discrimination and postprocessing steps. Cl. TPR and Cl. prec indicate cluster-wise TPR and cluster-wise precision respectively.

| **Steps** | **Cl. TPR** | **FPavg** |
|---|---|---|
| Cand. det. without FRST | 0.95 | 325.9 |
| Cand. det. using FRST | 0.95 | 25.1 |
| Cand. disc. using teacher model $T_c$ | 0.95 | 41.8 |
| Cand. disc. without KD | - | - |
| Cand. disc. using KD | 0.95 | 20.4 |
| After postproc. | - | - |

# 4 CROSS-VALIDATION ON UKBB AND OXVASC DATASETS: PERFORMANCE METRICS AT CLUSTER-WISE TPR OF 95%

**Table S4.** Cross-validation on the UKBB and OXVASC datasets: performance metrics at candidate detection, discrimination and post-processing steps. Cl. TPR and Cl. prec indicate cluster-wise TPR and cluster-wise precision respectively. C. det - candidate detection, C. disc - candidate discrimination.

| Datasets | Steps | Cl. TPR | FPavg |
|---|---|---|---|
| **UKBB (SWI)** | C. det. | 0.95 | 152.8 |
| | C. disc. | 0.95 | 24.2 |
| | Postproc. | - | - |
| **OXVASC (T2*-GRE)** | C. det. | 0.95 | 591.7 |
| | C. disc. | 0.95 | 48.4 |
| | Postproc. | - | - |

# 5 COMPARISON OF EXISTING PATCH-LEVEL CMB DETECTION METHODS

**Table S5.** Comparison of the performance of the existing methods that were developed and evaluated at patch-level. Pl.TPR - patch-level TPR, Pl.Prec - patch-level precision, Pl.Acc - patch-level accuracy.

| Methods | Sequence(s) (# test subjects) | Patch-size | # CMB/non-CMB patches | CMB patches detection performance |
|---|---|---|---|---|
| Zhang et al., 2016 [9] (10-fold cross-validation) | SWI (5) | 20 × 20 | 30,478/30,711 | Pl.TPR - 93.2%, Pl.acc - 93.22% |
| Chen et al., 2018 [10] | SWI (12) | 16 × 16 × 8 | 377/1235 | Pl.TPR - 94%, FPavg - 11.7, Cl.Prec - 72% |
| Zhang et al., 2018 [11] (10-fold cross-validation) | SWI (20) | - | 68,847/68,829 | Pl.TPR - 93.05%, Pl.acc - 93.06% |
| Zhang et al., 2018 [12] (10-fold cross-validation) | SWI (20) | 61 × 61 | 68,847/68,829 | Pl.TPR - 95.13%, Pl.acc - 94.23% |
| Hong et al., 2019 [13] (10-fold cross-validation) | SWI (20) | 61 × 61 | 4287/4287 | Pl.TPR - 95.7%, Pl.acc - 97.4% |
| Wang et al., 2019 [14] (10-fold cross-validation) | SWI (20) | 61 × 61 | 10000/10000 | Pl.TPR - 97.8%, Pl.acc - 97.7% |
| Lu et al., 2021 [15] (5-fold cross-validation) | SWI (20) | 41 × 41 | 6407 / 6624 | Pl.TPR - 98.27%, Pl.acc - 98.60% |

## REFERENCES

[1]Wei Bian, Christopher P Hess, Susan M Chang, Sarah J Nelson, and Janine M Lupo. Computer-aided detection of radiation-induced cerebral microbleeds on susceptibility-weighted MR images. *NeuroImage: clinical*, 2:282–290, 2013.

[2]Amir Fazlollahi, Fabrice Meriaudeau, Victor L Villemagne, Christopher C Rowe, Paul Yates, Olivier Salvado, and Pierrick Bourgeat. Efficient machine learning framework for computer-aided detection of cerebral microbleeds using the radon transform. In *2014 IEEE 11th international symposium on biomedical imaging (ISBI)*, pages 113–116. IEEE, 2014.

[3]Amir Fazlollahi, Fabrice Meriaudeau, Luca Giancardo, Victor L Villemagne, Christopher C Rowe, Paul Yates, Olivier Salvado, Pierrick Bourgeat, AIBL Research Group, et al. Computer-aided detection of cerebral microbleeds in susceptibility-weighted imaging. *Computerized Medical Imaging And Graphics*, 46:269–276, 2015.

[4]Babak Ghafaryasl, Fedde van der Lijn, Mariëlle Poels, Henri Vrooman, Mohammad Arfan Ikram, Wiro J Niessen, Aad van der Lugt, Meike Vernooij, and Marleen de Bruijne. A computer aided detection system for cerebral microbleeds in brain MRI. In *2012 9th IEEE international symposium on biomedical imaging (ISBI)*, pages 138–141. IEEE, 2012.

[5]Qi Dou, Hao Chen, Lequan Yu, Lin Shi, Defeng Wang, Vincent CT Mok, and Pheng Ann Heng. Automatic cerebral microbleeds detection from MR images via independent subspace analysis based hierarchical features. In *2015 37th annual international conference of the IEEE engineering in medicine and biology society (EMBC)*, pages 7933–7936. IEEE, 2015.

[6]Hao Chen, Lequan Yu, Qi Dou, Lin Shi, Vincent CT Mok, and Pheng Ann Heng. Automatic detection of cerebral microbleeds via deep learning based 3D feature representation. In *2015 IEEE 12th international symposium on biomedical imaging (ISBI)*, pages 764–767. IEEE, 2015.

[7]Qi Dou, Hao Chen, Lequan Yu, Lei Zhao, Jing Qin, Defeng Wang, Vincent CT Mok, Lin Shi, and Pheng-Ann Heng. Automatic detection of cerebral microbleeds from MR images via 3d convolutional neural networks. *IEEE transactions on medical imaging*, 35(5):1182–1195, 2016.

[8]Saifeng Liu, David Utriainen, Chao Chai, Yongsheng Chen, Lin Wang, Sean K Sethi, Shuang Xia, and E Mark Haacke. Cerebral microbleed detection using susceptibility weighted imaging and deep learning. *NeuroImage*, 198:271–282, 2019.

[9]Yu-Dong Zhang, Xiao-Xia Hou, Yi-Ding Lv, Hong Chen, Yin Zhang, and Shui-Hua Wang. Sparse autoencoder based deep neural network for voxelwise detection of cerebral microbleed. In *2016 IEEE 22nd International Conference on Parallel and Distributed Systems (ICPADS)*, pages 1229–1232. IEEE, 2016.

[10]Yicheng Chen, Javier E Villanueva-Meyer, Melanie A Morrison, and Janine M Lupo. Toward automatic detection of radiation-induced cerebral microbleeds using a 3d deep residual network. *Journal of digital imaging*, pages 1–7, 2018.

[11]Yu-Dong Zhang, Xiao-Xia Hou, Yi Chen, Hong Chen, Ming Yang, Jiquan Yang, and Shui-Hua Wang. Voxelwise detection of cerebral microbleed in cadasil patients by leaky rectified linear unit and early stopping. *Multimedia Tools and Applications*, 77(17):21825–21845, 2018.

[12]Yu-Dong Zhang, Yin Zhang, Xiao-Xia Hou, Hong Chen, and Shui-Hua Wang. Seven-layer deep neural network based on sparse autoencoder for voxelwise detection of cerebral microbleed. *Multimedia Tools and Applications*, 77(9):10521–10538, 2018.

[13]Jin Hong, Hong Cheng, Yu-Dong Zhang, and Jie Liu. Detecting cerebral microbleeds with transfer learning. *Machine Vision and Applications*, 30(7):1123–1133, 2019.

[14]Shuihua Wang, Chaosheng Tang, Junding Sun, and Yudong Zhang. Cerebral micro-bleeding detection based on densely connected neural network. *Frontiers in neuroscience*, 13:422, 2019.

[15]Si-Yuan Lu, Deepak Ranjan Nayak, Shui-Hua Wang, and Yu-Dong Zhang. A cerebral microbleed diagnosis method via featurenet and ensembled randomized neural networks. *Applied Soft Computing*, page 107567, 2021.
